# Targeted mutagenesis on PDGFRα-Fc identifies amino acid modifications that allow efficient inhibition of HCMV infection while abolishing PDGF sequestration

**DOI:** 10.1371/journal.ppat.1009471

**Published:** 2021-03-29

**Authors:** Svenja Feldmann, Immanuel Grimm, Dagmar Stöhr, Chiara Antonini, Peter Lischka, Christian Sinzger, Cora Stegmann

**Affiliations:** 1 Institute of Virology, Ulm University Medical Center, Ulm, Germany; 2 AiCuris Anti-infective Cures GmbH, Wuppertal, Germany; 3 Department of Molecular Medicine, University of Padua, Padua, Italy; Louisiana State University Health Sciences Center, UNITED STATES

## Abstract

Platelet-derived growth factor receptor alpha (PDGFRα) serves as an entry receptor for the human cytomegalovirus (HCMV), and soluble PDGFRα-Fc can neutralize HCMV at a half-maximal effective concentration (EC50) of about 10 ng/ml. While this indicates a potential for usage as an HCMV entry inhibitor PDGFRα-Fc can also bind the physiological ligands of PDGFRα (PDGFs), which likely interferes with the respective signaling pathways and represents a potential source of side effects. Therefore, we tested the hypothesis that interference with PDGF signaling can be prevented by mutations in PDGFRα-Fc or combinations thereof, without losing the inhibitory potential for HCMV. To this aim, a targeted mutagenesis approach was chosen. The mutations were quantitatively tested in biological assays for interference with PDGF-dependent signaling as well as inhibition of HCMV infection and biochemically for reduced affinity to PDGF-BB, facilitating quantification of PDGFRα-Fc selectivity for HCMV inhibition. Mutation of Ile 139 to Glu and Tyr 206 to Ser strongly reduced the affinity for PDGF-BB and hence interference with PDGF-dependent signaling. Inhibition of HCMV infection was less affected, thus increasing the selectivity by factor 4 and 8, respectively. Surprisingly, the combination of these mutations had an additive effect on binding of PDGF-BB but not on inhibition of HCMV, resulting in a synergistic 260fold increase of selectivity. In addition, a recently reported mutation, Val 242 to Lys, was included in the analysis. PDGFRα-Fc with this mutation was fully effective at blocking HCMV entry and had a drastically reduced affinity for PDGF-BB. Combining Val 242 to Lys with Ile 139 to Glu and/or Tyr 206 to Ser further reduced PDGF ligand binding beyond detection. In conclusion, this targeted mutagenesis approach identified combinations of mutations in PDGFRα-Fc that prevent interference with PDGF-BB but maintain inhibition of HCMV, which qualifies such mutants as candidates for the development of HCMV entry inhibitors.

## Author summary

Human cytomegalovirus is a major cause of congenital birth defects. Yet, currently the best way to avoid cytomegalovirus disease is to prevent infection of pregnant women through hygiene measures. Once the mother is infected there is no approved treatment to block transmission to the fetus. One intensively researched option is to neutralize the virus produced by the infected mother with anti-HCMV antibodies. Yet, as the efficiency of this approach remains to be demonstrated, alternative approaches need to be considered. Similar to antibodies, PDGFRα-Fc binds to the virus and blocks infection, but it is more potent and has a broader activity of inhibition which makes it a promising alternative. A problem however is that PDGFRα-Fc can not only bind to the virus but also to PDGFs which are important growth factors involved in cell-cycle regulation and tissue development. The results of this study offer a solution. Combinations of mutations were identified that can be introduced in PDGFRα-Fc to abrogate sequestration of PDGFs. Thus, the potential side effects of PDGFRα-Fc can be circumvented while it remains active against HCMV. These results pave the way for development of PDGFRα-Fc as a promising HCMV inhibitor.

## Introduction

Human cytomegalovirus (HCMV) is a ubiquitous pathogen that is found worldwide in 45–100% of the population [1]. Although the vast majority of infections is asymptomatic or mild, HCMV is the leading infectious cause of congenital birth defects in the western world and a continuous risk factor for transplant recipients. The currently available drugs inhibit replication and packaging of the viral genome and they are essential for successful transplantations [2,3]. Unfortunately, their use is in part limited by severe side effects and/or resistance [4], also none of them has been approved for prevention or treatment of intrauterine HCMV infection. Especially for prevention of congenital CMV, there is a continuing need for alternative treatment options.

One alternative strategy is to block virus entry, which has been successfully applied in anti-retroviral therapy [5]. HCMV is however different from HIV, as it can infect most cell types within the human body and contains a multitude of glycoproteins [6,7]. In addition to the core fusion machinery of herpesviruses which is composed of the fusogenic gB trimer and a complex formed by the glycoproteins H and L, HCMV encodes for additional proteins which mediate its broad cell tropism [8]. These accessory proteins form multimeric complexes with the conserved gH/gL complex [9–15]. The gH/gL/gO trimer of HCMV mediates cell-free infection independent of the cell type, while the pentameric complex gH/gL/pUL128/pUL130/pUL131A is additionally needed for infection of endothelial, epithelial and myeloid cells [16–23]. In recent years, several receptors for these glycoprotein complexes have been identified, including PDGFRα, NRP2, OR14I1 [7,24–27]. The best studied so far and the only one that was shown to interact with the gH/gL/gO trimer is the platelet-derived growth factor receptor alpha (PDGFRα), which is abundantly expressed on mesenchymal cells such as fibroblasts and trophoblasts [24–26,28–31]. The viral gH/gL/gO complex binds to PDGFRα expressed on the cell surface to mediate entry of virus particles [25,29,30]. We and others have demonstrated previously that a soluble PDGFRα-Fc fusion protein efficiently inhibits cell-free HCMV infection [25,26,29]. With a half-maximal effective concentration (EC50) of 10 ng/ml in fibroblasts, inhibition of fibroblast infection by PDGFRα-Fc is highly efficient as compared to neutralization by monoclonal antibodies [29,32–34]. This decoy receptor binds to gH/gL/gO on the virus particles and prevents them from binding to and penetrating into host cells [29]. Importantly, PDGFRα-Fc inhibits the infection of various cell types potently. For endothelial cells, in which infection does not rely on the interaction of gO with PDGFRα, the inhibitory effect of PDGFRα-Fc is due to inhibition of virus binding rather than penetration, indicating that gO is important for virus adsorption to these cells [22,29]. The efficient neutralization across different cell-types sets PDGFRα-Fc apart from many of the monoclonal antibodies that have been clinically tested because they are directed against components of the viral pentamer complex and inhibit infection of endothelial and epithelial cells more efficiently than infection of fibroblasts [32,35–37].

However, using PDGFRα-Fc as a decoy receptor against HCMV infection could potentially impair cellular signaling by sequestration of the natural ligands of PDGFRα. It has been shown that cellular PDGFRα interacts with PDGF-AA, PDGF-BB, PDGF-AB, PDGF-CC as well as VEGF and that soluble PDGFRα-Fc can additionally bind to PDGF-DD [38–40]. The interplay between platelet-derived growth factors (PDGFs) and their receptors PDGFRα and PDGFRβ plays an important role in development as well as in the regulation of various physiological processes like cell migration and proliferation in adults [40,41]. Hence, administering soluble PDGFRα-Fc could hypothetically cause side effects by sequestration of PDGFs and in consequence reduction of PDGF-dependent signaling. For further development of PDGFRα-Fc as an entry inhibitor, circumvention of PDGF sequestration is desirable. Mutation or deletion of the PDGF binding site in PDGFRα-Fc would be the most straight forward way to this aim. However, it has been shown that PDGFs can outcompete HCMV, indicating that the binding site of PDGFRα for the HCMV glycoprotein complex gH/gL/gO and the PDGFs are located in the same domains and might overlap to some extent [24,29,30,42]. In this study, a targeted mutagenesis approach was used to identify amino acid positions within the predicted ligand binding site of PDGFRα that are important for interaction with growth factors but dispensable for neutralization of the virus. The PDGFRα-Fc mutants were tested regarding affinity to PDGF, interference with cellular signaling and inhibition of HCMV infection. The mutations I139E, Y206S, V242K and combinations thereof abolished the blocking effect of PDGFRα-Fc on PDGF-dependent signaling while maintaining anti-HCMV activity. The results demonstrate that combinations of these point mutations drastically increased the selectivity for HCMV inhibition by loss of affinity to PDGF. These results open the possibility for development of soluble PDGFRα-Fc as an HCMV entry inhibitor.

## Results

### PDGFRα-Fc inhibits gH/gL/gO-dependent infection at low concentrations independent of the cell type

**Fig 1:**
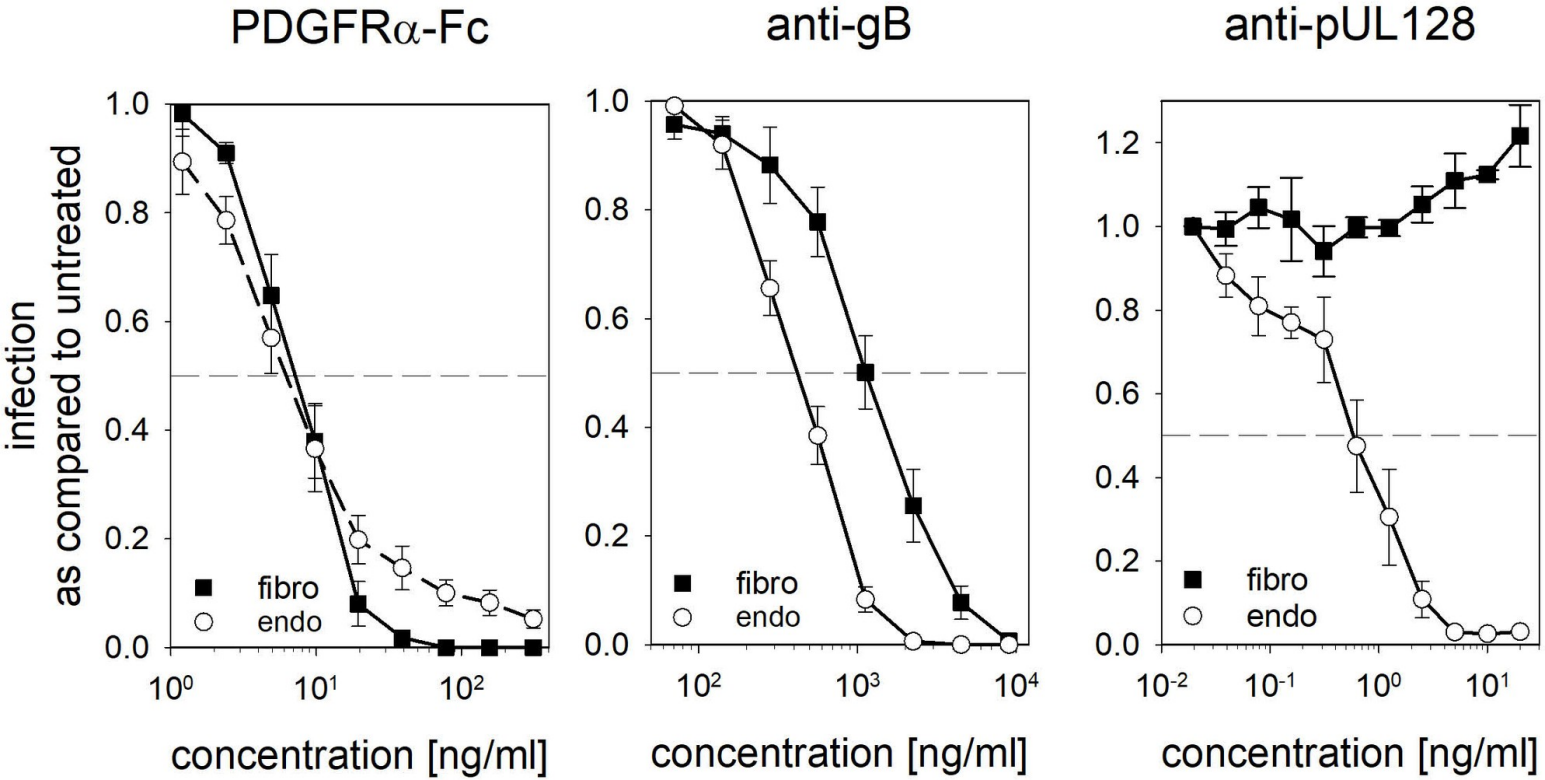
Comparison of the inhibition of fibroblast and endothelial cell infection by PDGFRα-Fc and anti-gB, anti-pUL128 antibodies. Neutralization of TB40-BAC4 infection of fibroblasts (fibro) and endothelial cells (endo) by PDGFRα-Fc, anti-gB clone C23 and anti-pUL128 clone 4I22 was tested. The virus was diluted to an MOI of ≤ 1 and preincubated for 2 h at 37°C with the indicated concentrations of PDGFRα-Fc or the respective purified monoclonal antibody. The cells were incubated with the virus/inhibitor mixtures for 2 h. After 24h the cells were fixed and stained for the viral immediate early proteins to determine the percentage of infected cells in each condition. Shown is the percentage of infection as compared to an untreated control. The experiments were performed 5 times for PDGFRα-Fc, 7 times for anti-gB and 3 times for anti-pUL128. Error bars indicate standard error of the mean.

We have previously reported that PDGFRα-Fc inhibits both trimer and pentamer-dependent HCMV infection with similarly high efficiency. This can be explained by the importance of the gH/gL/gO complex for cell-free infection independent of the cell type. In theory, equal efficiency could be expected from antibodies targeting viral proteins that are essential for infection of all cell types, such as gB. However, many studies have shown that infection of fibroblasts is more resistant to inhibition by such antibodies than that of endothelial/epithelial cells [33,43,44]. This apparent difference between our results with PDGFRα-Fc and the published data on antibody neutralization could be due to differences in the target cells or the experimental procedures. Therefore, a well characterized anti-gB antibody was tested in the same experimental system as PDGFRα-Fc (Fig 1). For inhibition of pentamer-dependent infection of endothelial cells, a highly potent anti-pUL128 monoclonal was included as an additional reference. HCMV TB40-BAC4 was diluted to an MOI ≤ 1 (resulting in less than 64% infection) and preincubated for 2 h at 37°C with various concentrations of the soluble gO receptor PDGFRα-Fc, anti-gB clone C23 or anti-pUL128 clone 4I22. The virus/inhibitor mixture was then incubated with either fibroblasts or endothelial cells for 2 hours. One day after infection, the cells were fixed and stained for viral immediate-early antigens to determine the percentage of infected cells. As expected, the anti-pUL128 antibody inhibited infection of endothelial cells very potently, with a half-maximal effective concentration (EC50) of about 1 ng/ml but did not reduce infection of fibroblasts. The anti-gB antibody C23 inhibited infection of both cell-types, but with varying efficiency. In endothelial cells, 50% inhibition was achieved at 500 ng/ml, while the same level of inhibition on fibroblasts required 2000 ng/ml. In contrast, PDGFRα-Fc inhibited both cell types equally efficient with an EC50 of about 10 ng/ml. These results correspond well to previous findings with neutralizing antibodies and indicate that the indiscriminate, high efficiency of inhibition of HCMV infection by PDGFRα-Fc may be a rather unique property of PDGFRα-Fc.

### Deletions of the predicted PDGF binding sites in PDGFRα-Fc differentially affect HCMV inhibition

**Fig 2:**
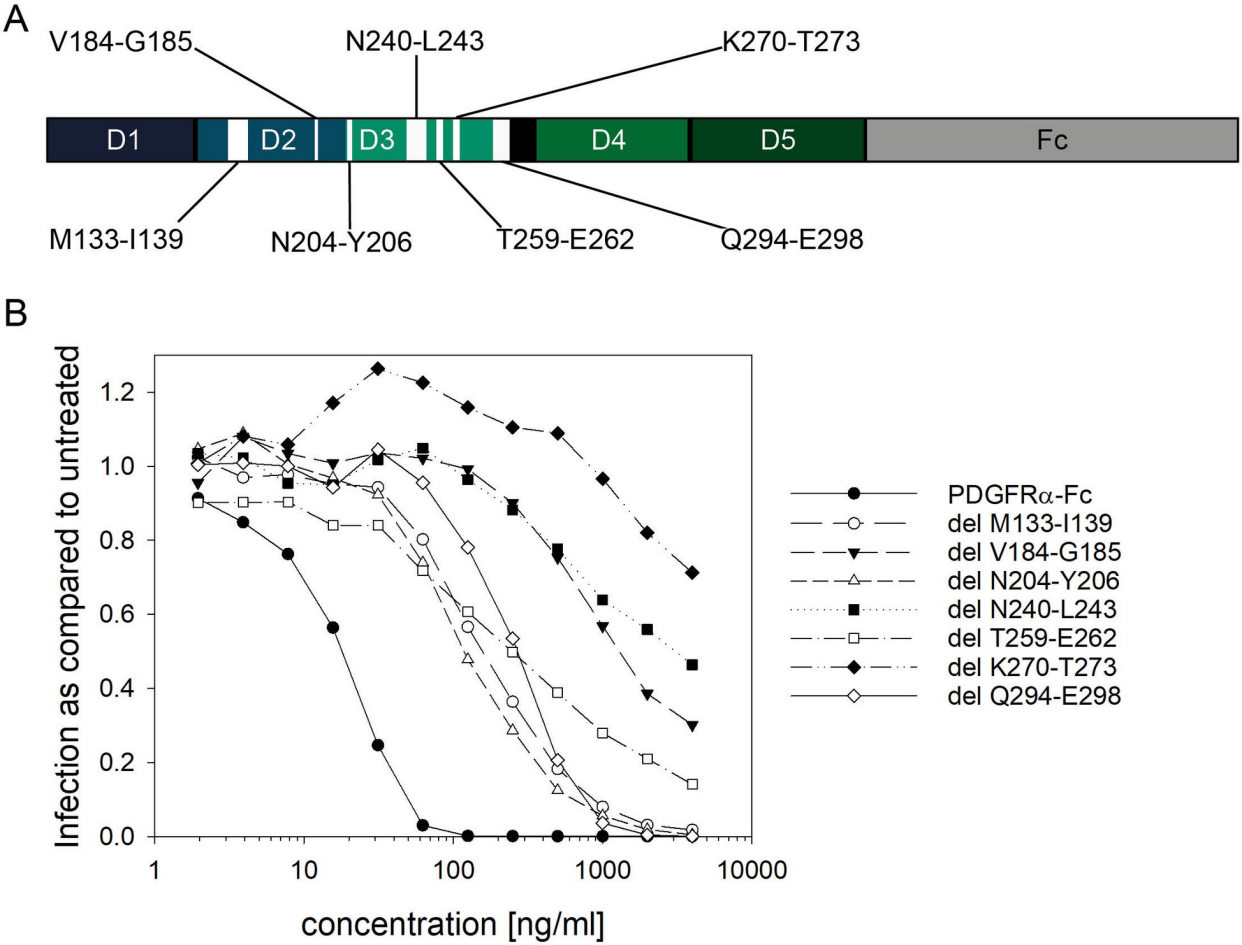
Screening of deletions in the predicted ligand binding sites of PDGFRα-Fc for their ability to inhibit HCMV infection. **A**: Overview of the relative locations of the predicted ligand binding sites in domains 2 and 3 of PDGFRα-Fc. Position numbers are based on the amino acid sequence of cellular PDGFRα (XP_005265800). **B**: The effect of PDGFRα-Fc wild type and deletion mutants on HCMV infection of fibroblasts was tested. Different concentrations of the soluble receptor variants were preincubated with an MOI of 1 of HCMV TB40-BAC4-IE-GLuc reporter virus. Expression of GLuc in samples treated with the different PDGFRα-Fc variants were compared to cells incubated with untreated virus. Dose response curves were generated as an average of 3 independent experiments using 2 different preparations of the luciferase expressing TB40-BAC4-IE-GLuc reporter virus and 2 different protein preparations of the soluble receptors.

The major drawback of PDGFRα-Fc for use against HCMV transmission is that this soluble receptor can be expected to bind to the ligands of PDGFRs, thereby interfering with cellular signaling. A way to solve this potential problem could be to modify PDGFRα-Fc in a way that increases its selectivity for HCMV by diminishing interaction with PDGFs. We hypothesized that it is possible to separate HCMV inhibition from PDGF binding by targeted mutation of specific amino acids that are involved in binding to PDGFs. To date, no high-resolution structure of PDGFRα bound to HCMV gO has been obtained and also the structure of PDGFRα bound to PDGFs has not been solved. However, the interactions of PDGF-AA and PDGF-BB with PDGFRβ are well studied and their structures have been solved [45,46]. PDGFRα and PDGFRβ have 30% sequence identity and they have overlapping activities for PDGFs [46]. Therefore, a computational prediction by Torrente and colleagues [47] was utilized as a starting point for the identification of sites in PDGFRα that are essential for ligand binding but dispensable for HCMV inhibition. In this study, the PDGF-AA and PDGF-BB binding sites in PDGFRα were predicted based on the previously published structure of PDGFRβ bound to PDGF-BB [46]. As a result of their docking simulations, Torrente et al. provided a list of amino acids in the extracellular domain of PDGFRα that are likely to be important for binding to PDGF-AA and PDGF-BB [47]. To narrow down the list of promising sites for reduction of ligand binding, a set of seven small deletions was introduced at the predicted sites in domains two and three of PDGFRα in the background of the Fc fusion vector pFuse-PDGFRα-ECD-hIgG1-Fc2 (Fig 2A, [42]). The soluble receptor mutants were expressed in 293F cells and affinity purified using Protein A. To identify sites which are dispensable for binding to HCMV, the capacity of the PDGFRα-Fc deletion mutants to inhibit HCMV infection was assessed with a standard neutralization assay utilizing a Gaussia luciferase reporter virus (TB40-BAC4-IE-GLuc, [48]). Cell- and luciferase-cleared preparations of the virus were diluted to an MOI of ≤ 1 and preincubated with various concentrations (4,000 to 1 ng/ml) of purified PDGFRα-Fc. After an incubation period of 2 hours at 37°C, the virus was incubated with the fibroblasts for 2 hours. One day later, the luciferase-containing medium was collected and the Gaussia activity therein was measured. The extent of infection initiated by the pretreated virus was quantified relative to untreated control samples. Wild type PDGFRα-Fc inhibited HCMV at a half-maximal effective concentration (EC50) of <20 ng/ml and fully blocked HCMV inhibition already at 100 ng/ml (Fig 2B). All deletions reduced the inhibitory effect of PDGFRα-Fc to some extent, as indicated by a shift of the dose-response towards higher concentrations (Fig 2B). With four out of seven mutations a concentration of 4,000 ng/ml was insufficient to achieve full inhibition. As this indicated sensitivity of PDGFRα-Fc to disruptions at these sites, they were deemed less promising as a target for further mutagenesis. In contrast, PDGFRα-Fc deletion mutants delM133-I139, delN204-Y206 and delQ294-E298 completely blocked HCMV entry at 2000 ng/ml, with EC50s about 10fold higher than that of wild type PDGFRα-Fc. These results demonstrated that mutations at these sites are less disruptive for overall protein function and are compatible with neutralization of the virus. Therefore, this smaller set of predicted PDGF binding sites was used as a basis for a further scanning of individual amino acid positions that would allow for disruption of PDGF binding without loss of virus neutralization.

### Mutations of isoleucine 139 and tyrosine 206 in PDGFRα-Fc reduce sequestration of PDGF-BB

**Fig 3:**
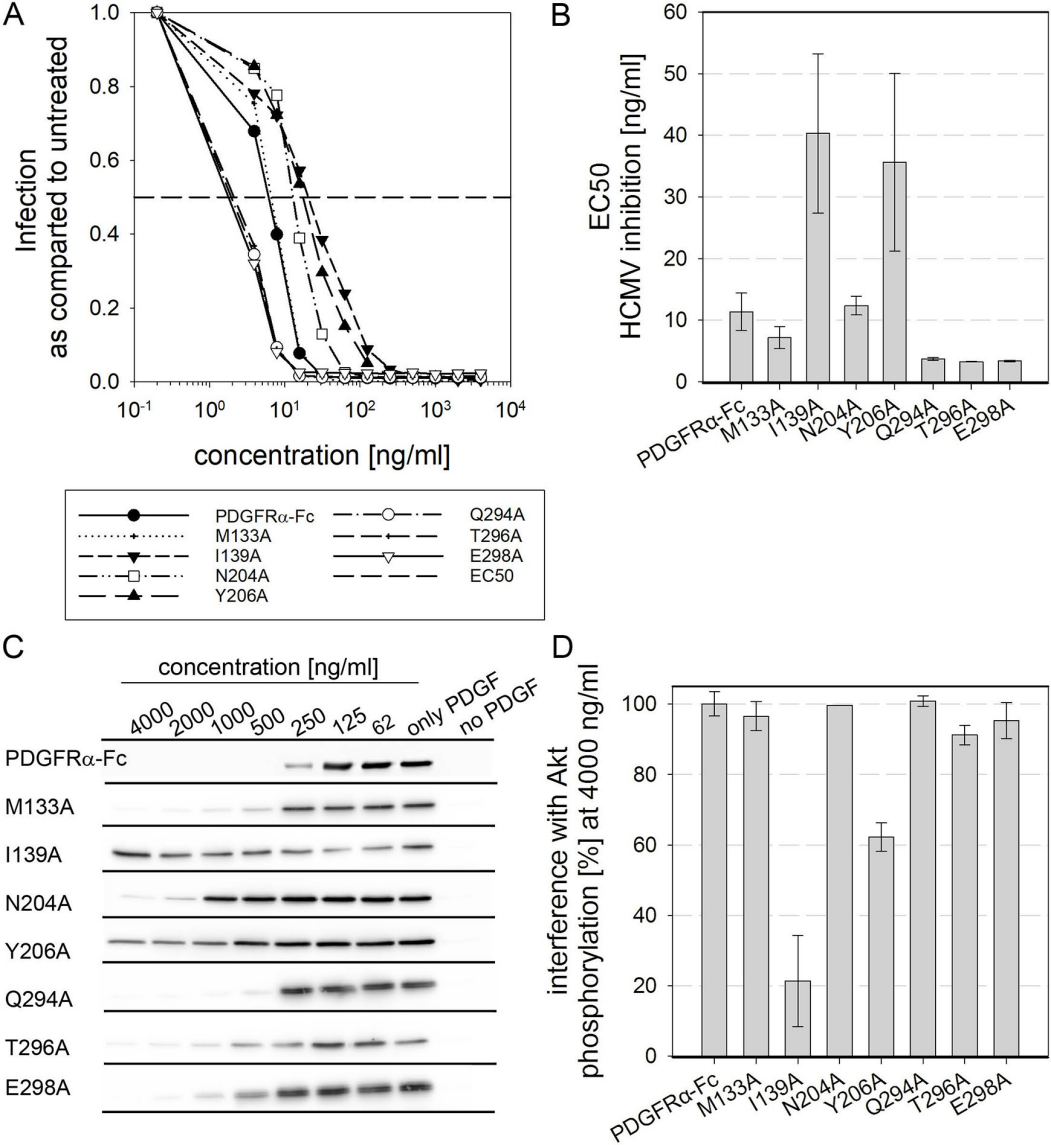
Mutation of isoleucine 139 and tyrosine 206 reduces sequestration of PDGF-BB. **A + B:** Quantification of HCMV inhibition by PDGFRα-Fc alanine exchange mutants was assessed by preincubation of the HCMV TB40-BAC4-IE-GLuc reporter virus with PDGFRα-Fc derivatives before infection of fibroblasts. The degree of infection was measured relative to a virus only condition. **A** depicts the average dose-response curves as calculated from at least 3 independent experiments. **B:** The individual dose-response curves from each experiment were used as a basis to calculate the half-maximal effective concentrations (EC50s) needed for HCMV inhibition. **C + D:** Biological interference of PDGFRα-Fc alanine exchange mutants with PDGF-dependent signaling was tested by preincubation of 50 ng/ml PDGF-BB with various concentrations of PDGFRα-Fc derivatives before stimulation of fibroblasts. Immunoblot staining of Phospho-Akt served as a measure for PDGF induced signaling. **C** shows immunoblot examples and **D** summarizes the interference of 4000 ng/ml of the different PDGFRα-Fc variants with Akt phosphorylation. All signals were normalized to actin to control for consistent loading. 100% interference equals complete inhibition of Akt phosphorylation to the level of cells that did not receive PDGF-BB. 0% interference corresponds to full signal intensity to the level of cells stimulated with untreated PDGF-BB. Summarized are experiments using two independent preparations of the soluble receptors. Error bars indicate standard error of the mean.

For this second screening step, we considered only amino acids that were a) included in deletions that were compatible with full inhibition of HCMV infection and b) predicted to be involved in binding of PDGF-A and PDGF-B. These amino acids were M133, I139, N204, Y206, Q294, T296 and E298. An alanine exchange approach was chosen in order to avoid gross structural changes in the protein. The seven alanine exchange mutations were introduced into PDGFRα-Fc and the purified soluble receptor mutants were tested regarding their ability to inhibit HCMV infection. The shift in dose-dependent inhibition of HCMV by these more subtle mutations was less pronounced as compared to those of the respective deletions (Fig 3A and 3B and Table 1). Notably, both slightly enhanced and slightly reduced HCMV neutralization was observed. All three mutations in the peptide site 294–298 increased the inhibitory effect of PDGFRα-Fc about 3fold. On the other side, mutation of isoleucine 139 and tyrosine 206 decreased the efficiency of HCMV inhibition by about 4fold. Mutation of asparagine 204 and methionine 133 did not change the EC50. Overall, the impact on the HCMV neutralization capacity of PDGFRα-Fc was mild for all alanine exchange mutants. The next step was to test whether any of those mutations would reduce interference with cellular signaling. To assess the risk of such a potential side effect, the impact of soluble PDGFRα-Fcs on PDGF-dependent signaling in cells was measured by an immunoblot based assay. PDGFRα can bind to a variety of different growth factors including PDGF-A, PDGF-B and PDGF-C [40,41], which will start signaling cascades that include phosphorylation of Akt. Therefore, the effect of soluble PDGFRα-Fc on Akt phosphorylation was chosen as a downstream measure of changes in binding to growth factors. The experiments were performed with PDGF-BB as the cellular response to PDGF-BB has been shown to be more pronounced than to PDGF-AA or -CC [49]. A concentration of 50 ng/ml PDGF-BB was used based on the manufacturer´s information that this concentration is sufficient to induce full fibroblast proliferation. Different concentrations of PDGFRα-Fc wild type and mutants were preincubated with dimeric PDGF-BB for 2 hours at 37°C before stimulation of fibroblasts. To reduce background phosphorylation, the cells were serum-starved prior to addition of the PDGF-BB/PDGFRα-Fc mixtures. After stimulation for 90 min, the cells were lysed and Akt phosphorylation was determined in immunoblots (Fig 3C). We observed that those mutations which reduced inhibition of HCMV also resulted in a reduced sequestration of PDGF-BB. At the highest concentration tested in these experiments (4000 ng/ml), wild type as well as five of the seven alanine-exchange mutants caused full interference. PDGF-dependent phosphorylation of Akt was reduced close to background level (Fig 3D). In contrast, preincubation of PDGF-BB with 4,000 ng/ml of PDGFRα-Fc I139A and Y206A reduced Akt phosphorylation by only 20% and 60%, respectively. These results demonstrated that mutation of isoleucine (I) 139 and tyrosine (Y) 206 mitigate the negative effect of PDGFRα-Fc on Akt phosphorylation. It can be concluded that both mutations I139A and Y206A reduce the sequestration of PDGFs by PDGFRα-Fc.

**Table 1:** Overview of HCMV inhibition by PDGFRα-Fc I139 and Y206 mutants.

	HCMV inhibition EC50 [ng/ml] (SEM)
PDGFRα-Fc wild type	5.6 (± 0.88)
PDGFRα-Fc I139E	32.8 (± 8.31)
PDGFRα-Fc I139L	6.6 (± 0.53)
PDGFRα-Fc I139V	9.5 (±1.26)
PDGFRα-Fc Y206F	5.9 (±0.94)
PDGFRα-Fc Y206S	31.1 (±3.70)
PDGFRα-Fc I139A + Y206A	19.8 (±0.78)
PDGFRα-Fc I139E + Y206S	25.2 (±1.34)

Please note: 10 ng/ml of PDGFRα-Fc correspond to 60 pmol/l

### Selected mutations at positions 139 and 206 of PDGFRα improve the interaction profile of the soluble receptor

To identify amino acid exchanges that would reduce PDGF sequestration the most and HCMV inhibition the least, hypothesis driven mutations at positions 139 and 206 were introduced into PDGFRα-Fc. We assumed that PDGF binding should be disrupted by changing either size or charge of respective side chains, since the strong affinity of PDGFRα to its PDGF ligands seems to involve hydrophobic interactions paired with an electrostatic network of hydrogen bonds [47,50,51]. However, whether those mutations would differentially affect PDGF binding and HCMV neutralization was not predictable. Therefore, we chose to investigate the impact of relatively small modifications regarding size (I139L, I139V) and polarity (Y206F) and changed the hydrophobicity of respective binding regions (I139E, Y206S).

**Fig 4:**
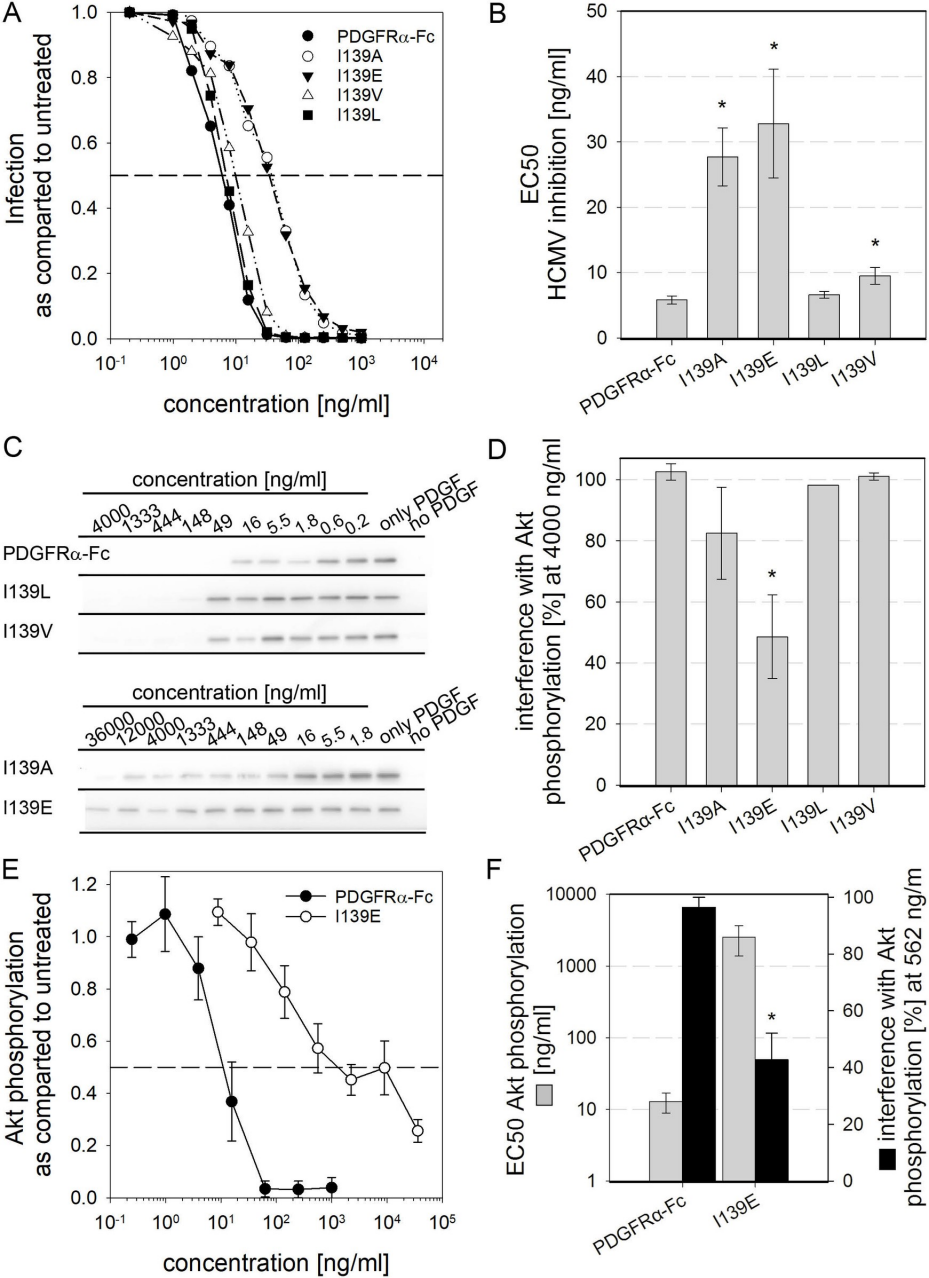
Replacement of isoleucine 139 with glutamic acid improves the inhibition profile of PDGFRα-Fc. **A + B:** Quantification of HCMV inhibition by the PDGFRα-Fc I139 mutants. TB40-BAC4-IE-GLuc at an MOI ≤ 1 was preincubated with different concentrations of PDGFRα-Fc derivatives before infection of fibroblasts. After 1 day the cells were fixed and stained for the viral immediate early antigens and the percentage of infected cells was determined. Dose-response curves showing the percent of infection relative to a virus only control (**A**) were used as a basis to calculate the half-maximal effective concentrations (**B**). Shown are the means of 5 independent experiments. **C to F:** Biological interference of PDGFRα-Fc I139 mutants with PDGF-dependent signaling was tested by preincubation of 6 ng/ml PDGF-BB with different concentrations of PDGFRα-Fc before stimulation of fibroblasts. **C + D:** PDGF-dependent signaling was assessed by staining for Phospho-Akt. **C** shows immunoblot examples and **D** summarizes the change in phospho-Akt signals after pretreatment of PDGF-BB with 4000g/ml PDGFRα-Fc. 100% interference equals complete inhibition of Akt phosphorylation to the level of cells that did not receive PDGF-BB. **E + F:** PDGF-dependent signaling was quantified with a phospho-Akt ELISA. The dose-response curves shown in **E** depict the mean of 3 independent experiments and **F** shows the EC50s calculated thereof in grey. The black bars depict the change in Akt phosphorylation when PDGF-BB was pretreated with 562 ng/ml of PDGFRα-Fc wild type or I139E. 100% interference equals complete inhibition of Akt phosphorylation to the level of cells that not receive PDGF-BB. All error bars indicate standard error of the mean and all experiments were performed with at least 2 independent protein preparations. Asterisks indicate statistically significant differences as compared to PDGFRα-Fc wild type determined by unpaired t-tests.

To evaluate the effects of these single amino acid exchanges on the interaction of soluble PDGFRα-Fc, the recombinant proteins were expressed and tested regarding their ability to inhibit HCMV infection and their impact on PDGF-dependent signaling (Fig 4). For measurement of HCMV inhibition the virus was preincubated with the soluble receptors before incubation with the cells. To obtain even more precise data, infection was measured by immunofluorescence staining for the viral immediate early antigens instead of by luciferase activity. Replacement of isoleucine 139 with leucine or valine had no or only a marginal effect as compared with wild type PDGFRα-Fc (Fig 4A and 4B and Table 1). The replacement with glutamic acid decreased the efficiency of HCMV inhibition 6fold, comparable to the alanine exchange mutant. For measurement of the effect on PDGF sequestration, the protocol of the assay was also adjusted to obtain higher sensitivity which was important because the alanine exchange mutants had already reduced PDGF sequestration close to the limits of detection. As a basis for optimization, the dose-response relationship of cellular Akt phosphorylation after treatment of cells with different concentrations of PDGF-BB was determined (S1 Fig). A concentration of 6 ng/ml PDGF-BB was found to result in strong Akt phosphorylation below saturation, increasing the assays sensitivity by limiting the amount of stimulant. It is important to note that consistently strong Akt phosphorylation after treatment with 6 ng/ml PDGF-BB was only achieved when the time of stimulation was reduced from 1.5 hours to 15 min, possibly due to cellular feedback mechanisms. This improved protocol was used to test the I139 mutants regarding their inhibitory effect on PDGF-dependent Akt phosphorylation (Fig 4C and 4D). The results of these analyses showed that replacement of I139 with glutamic acid decreased PDGF sequestration even beyond the effect of the alanine exchange mutation. In detail, treatment of 6 ng/ml PDGF-BB with 4000 ng/ml of the PDGFRα-Fc I139A mutant reduced the signal intensities of p-Akt in quantitative immunoblots by about 80% whereas I139E caused only about 50% reduction in signaling. To validate the results obtained by the phospho-Akt immunoblots, the impact of the soluble receptors on cellular signaling was additionally tested with a phospho-Akt ELISA. The pretreatment of PDGF-BB was performed as for the immunoblot readout. With this assay, dose-response curves for the impact of the PDGFRα-Fc fusion proteins on PDGF-dependent phosphorylation of Akt were generated (Fig 4E). While wild type PDGFRα-Fc blocked Akt phosphorylation at a concentration of 60 ng/ml, the I139E mutation decreased PDGF sequestration so that full inhibition of Akt phosphorylation was not even observed at 36,000 ng/ml. It is also noteworthy that the slope of the curve for the PDGFRα-Fc I139E mutant was much more gradual, with a lower Hill coefficient, indicating that the dynamics of the competition between the soluble and the cellular receptors for the ligands has changed profoundly. In line with this, the EC50 of I139E for inhibition of PDGF signaling was significantly increased by 2 log steps from 13 ng/ml for the wild type molecule to about 2000 ng/ml for the I139E mutant (Fig 4F). However, these numbers have to be interpreted carefully, due to the incomplete inhibition that precludes precise calculation of the EC50 for I139E. We also compared the effect of the PDGFRα-Fc variants on Akt phosphorylation at 562 ng/ml. This concentration is sufficient for full HCMV inhibition by PDGFRα wild type and I139E and therefore reflects a plausible concentration to aim at. PDGF-dependent Akt phosphorylation was almost completely blocked upon pretreatment with 562 ng/ml wild type PDGFRα-Fc, but only about 50% reduced with the I139E mutant. This demonstrated that I139E reduced PDGF sequestration but still interferes with cellular signaling.

Taken together, these results demonstrate that introducing a charge at position 139 by replacing isoleucine with glutamic acid is suitable to increase the selectivity index of PDGFRα-Fc. Mutation at this position decreases the ability of PDGFRα-Fc to sequester PDGFs about 100fold while inhibition of HCMV is only reduced 6fold.

**Fig 5:**
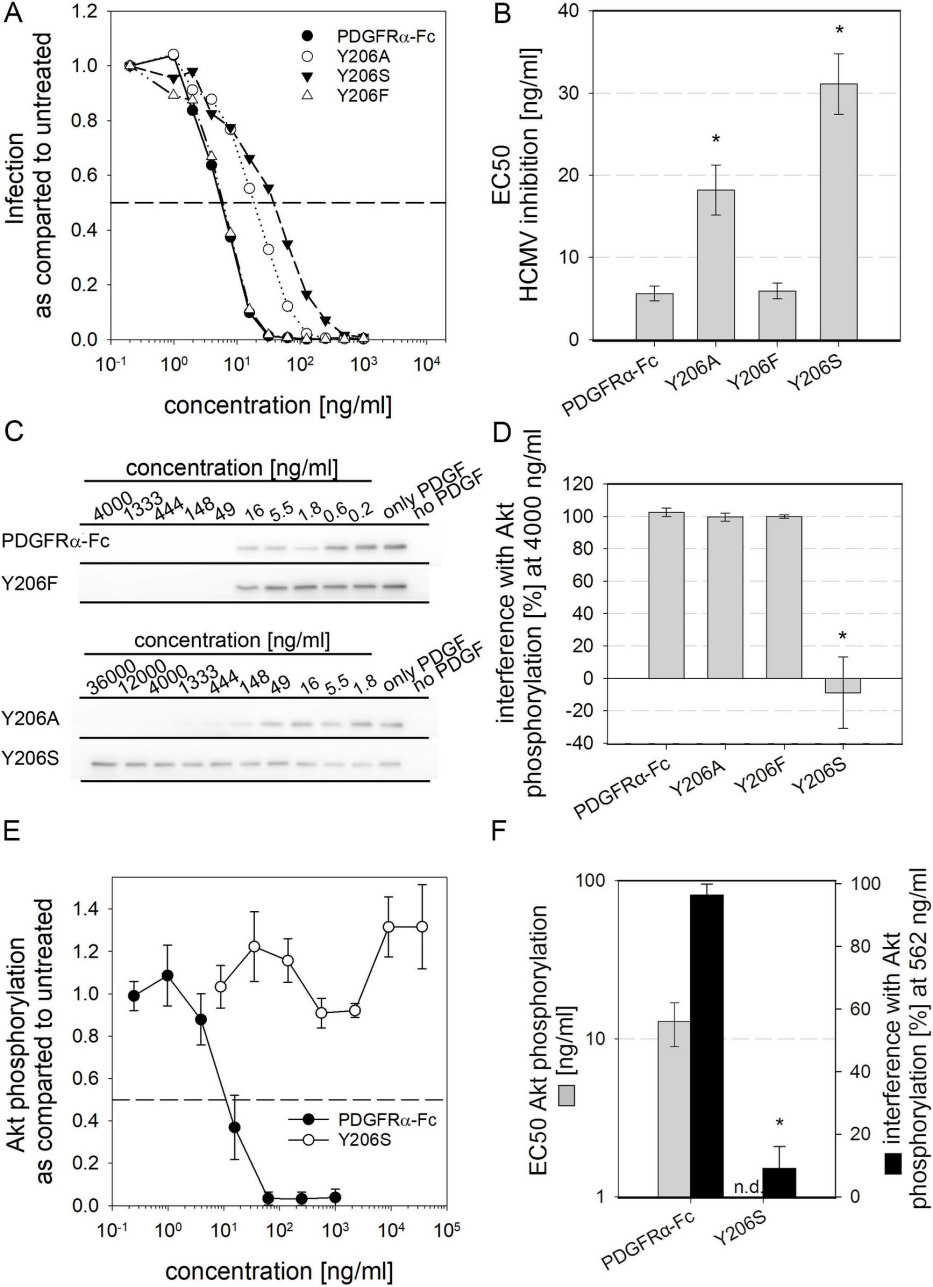
Replacement of tyrosine 206 with serine abolishes PDGF sequestration while maintaining HCMV inhibition. **A + B:** HCMV at an MOI ≤ 1 was pretreated with various concentrations of PDGFRα-Fc fusion proteins before infection of fibroblasts. Shown is the number of cells expressing the viral immediate early antigens relative to a virus only condition. Dose-response curves, as shown averaged in **A**, were used as a basis to calculate the half-maximal effective concentrations (**B**) in 3 independent experiments. **C to F:** Biological interference with PDGF-dependent signaling was tested by preincubation of 6ng/ml PDGF-BB with various concentrations of PDGFRα-Fc mutants or wild type before stimulation of fibroblasts. **C to D:** Staining of Phospho-Akt served as a measure for PDGF induced signaling. **C** shows immunoblot examples and **D** summarizes the interference of different PDGFRα-Fc variants with Akt phosphorylation. 100% interference equals complete inhibition of Akt phosphorylation and negative values indicate enhanced Akt phosphorylation as compared to cells that were stimulated with untreated PDGF-BB. **E +F:** PDGF-dependent signaling was quantified using a phospho-Akt ELISA. The dose-response curves shown in **E** depict the mean of 3 independent experiments and **F** shows the EC50s calculated thereof (grey bars) as well as the interference of 562 ng/ml of PDGFRα-Fc variants with PDGF-dependent Akt phosphorylation (black bars). N.d. stands for not determined and indicates that in this case no EC50 could be calculated due to lack of response. All error bars indicate standard error of the mean and all experiments were performed with at least 2 independent protein preparations. Asterisks indicate statistically significant differences as compared to PDGFRα-Fc wild type determined by unpaired t-tests.

The alanine exchange scanning had identified tyrosine 206 as another important site for interaction with PDGF-B. As described for the I139 mutations, the Y206F and Y206S PDGFRα-Fc mutants were generated and analyzed regarding inhibition of HCMV and interference with PDGF-dependent signaling. Pretreatment of HCMV with PDGFRα-Fc Y206F resembled the effect seen with the wild type protein, with regard to the overall dose response and the EC50 (Fig 5A and 5B and Table 1). Exchange of tyrosine with serine (Y206S) reduced the EC50 for HCMV inhibition by 6fold as compared to wild type, slightly more than the 4fold reduction caused by the alanine exchange at position 206. The same tendencies, yet to a much greater extent were also observed when the effect of those mutations on PDGF sequestration was measured (Fig 5C–5F). Y206F did not improve interference as compared to the alanine exchange mutant, in contrast Y206S significantly decreased the inhibition of PDGF-dependent cellular signaling as compared to wild type and the alanine mutant. No inhibition of Akt phosphorylation at all was observed even at 36,000 ng/ml. Sequestration of PDGF-BB was also below detection limit when Akt phosphorylation was quantified by ELISA, indicating strongly reduced, but not necessarily abolished PDGF-BB binding. It is noteworthy that PDGFRα-Fc Y206S, when applied at very high concentrations (greater than 4,000 ng/ml), slightly enhanced cellular signaling. Comparison at lower concentrations, that are more likely to be used against HCMV (i.e. 562 ng/ml), however allowed to quantify the difference between PDGFRα-Fc wild type and mutant. PDGFRα-Fc Y206S caused almost no reduction in Akt phosphorylation whereas wild type led to near complete inhibition of cellular signaling. As no actual dose dependent inhibition of cellular signaling was observed, no EC50s could be determined for this PDGF sequestration by PDGFRα-Fc. These results demonstrate that loss of the phenyl ring at position 206 of PDGFRα-Fc strongly impairs the inhibition of cellular signaling while the efficiency of HCMV inhibition was only slightly reduced.

### Combination of the most promising mutations further decreases PDGF sequestration

**Fig 6:**
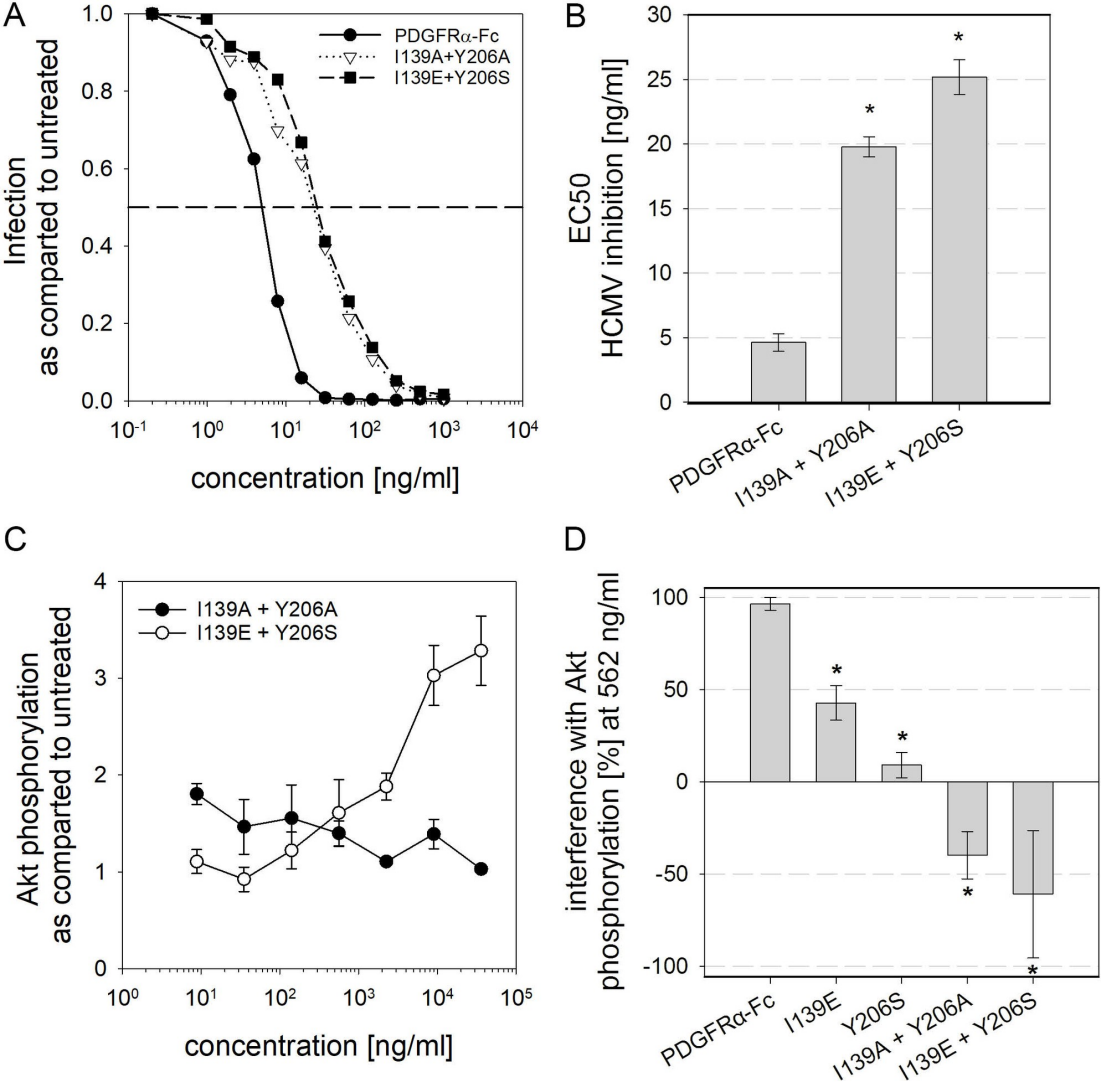
Combination of multiple amino acid exchanges in PDGFRα further reduces inhibition of PDGF dependent signaling. **A + B:** Quantification of HCMV inhibition by PDGFRα-Fc double amino acid exchange mutants. To measure the inhibition of infection, the rate of immediate early antigen-positive cells after infection with HCMV untreated or pretreated with PDGFRα-Fc was measured. An average of 3 independent dose-response experiments is shown in **A**. The corresponding EC50 values are shown in **B**. **C + D**: PDGF-dependent signaling after preincubation of 6 ng/ml PDGF-BB with various concentrations of PDGFRα-Fc mutants was quantified with a phospho-Akt ELISA. The dose-response curves shown in **C** depict the mean of 3 independent experiments and **D** compares the interference of 562 ng/ml of the different PDGFRα-Fcs with PDGF-dependent cellular signaling. 100% interference equals complete inhibition of Akt phosphorylation to the level of unstimulated cells whereas negative values indicate enhanced Akt phosphorylation as compared to cells that were stimulated with untreated PDGF-BB. All error bars indicate standard error of the mean.

After having established that mutations I139E and Y206S both significantly decrease PDGF sequestration while having only minor impacts on HCMV inhibition, we wanted to test whether safety of PDGFRα-Fc could be further improved by combining these mutations. Therefore, two more variants of PDGFRα-Fc were generated, a double alanine-exchange mutant PDGFRα-Fc I139A + Y206A and a PDGFRα-Fc I139E + Y206S double mutant. The double mutants were analyzed using the established assays for inhibition of HCMV and interference with PDGF-dependent cellular signaling (Fig 6). Quantification of HCMV inhibition revealed that both combinatory mutations slightly decreased the inhibitory effect on HCMV as compared to wild type PDGFRα-Fc, but not as compared to the respective single amino acid exchange mutants (Fig 6A and 6B and Table 2). Double alanine exchange resulted in a 4fold increased EC50 for HCMV inhibition as compared to wild type while combination of I139E and Y206S resulted in a 5fold increase, to 20 and 25 ng/ml, respectively. In both cases the increase of the EC50 for inhibition of HCMV was very similar as compared to the individual mutations which resulted in about 6fold increased EC50s (compare Fig 6B with Figs 4B and 5B). Because gO differs greatly between HCMV strains, inhibition of other HCMV strains by PDGFRα-Fc I139E + Y206S was also tested. While differences in the efficiency of neutralization of strains VHL/E, AD169, Towne and Merlin were observed for the PDGFRα-Fc mutant, infection of all strains was completely inhibited at 1000 ng/ml (S2 Fig). Additionally, combination of I139E and Y206S mutations did not reduce protein yield nor change protein quality (S1 Table and S3 Fig). When tested for inhibition of PDGF-dependent cellular signaling (Fig 6C and 6D), both double mutants did not inhibit Akt phosphorylation at all, even when applied at 36,000 ng/ml. It was noticeable however that while the I139A + Y206A mutant did not have any effect on cellular signaling, the I139E + Y206S mutant induced an increase in signaling when used at high concentrations, a phenomenon that was already observed for the Y206S single amino acid exchange mutant.

**Fig 7:**
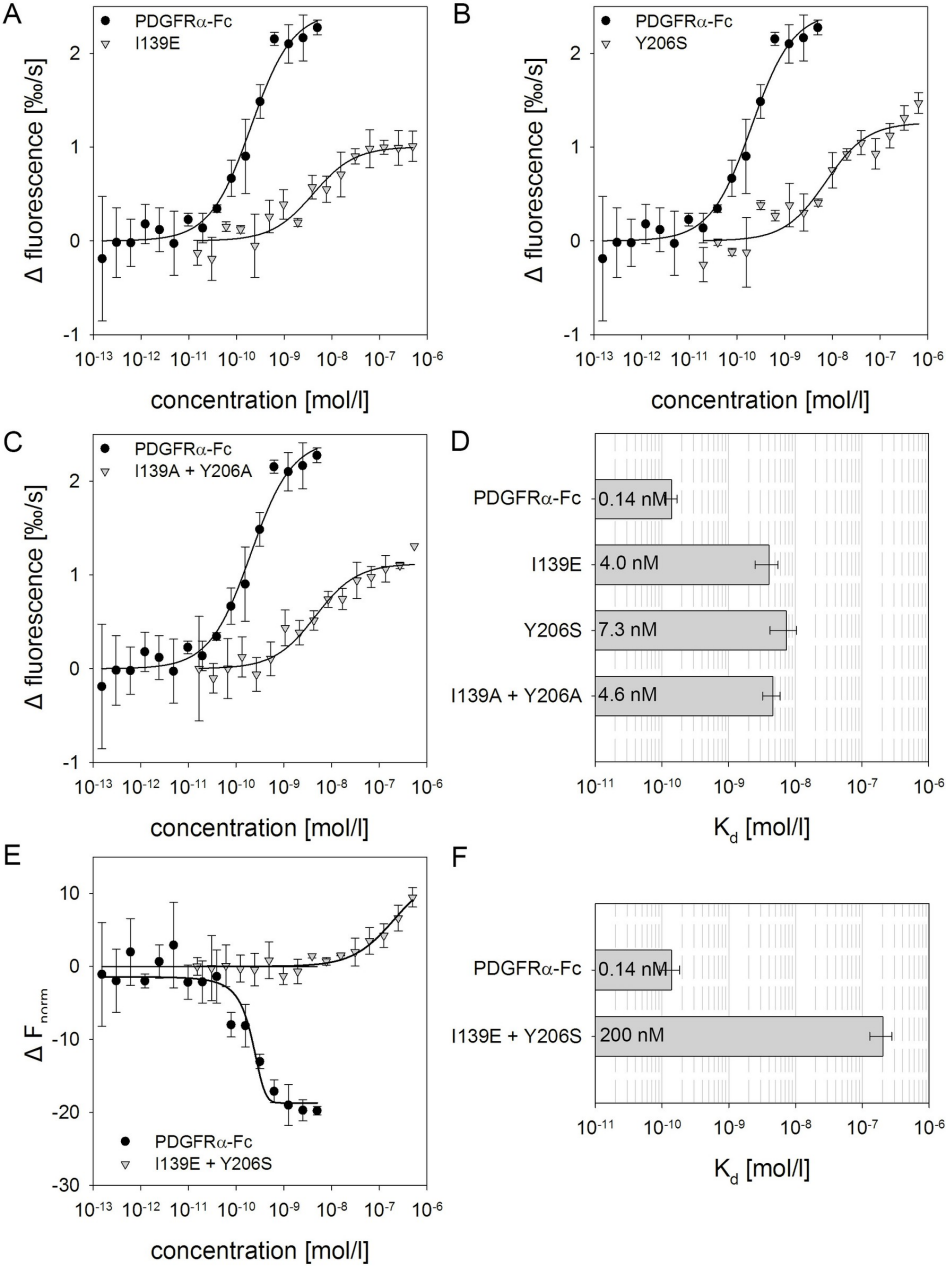
Mutations in PDGFRα-Fc reduce binding affinity for PDGF-BB. Quantification of biochemical binding affinity of PDGFRα-Fc variants for PDGF-BB was assessed by microscale thermophoresis. All experiments were performed with 0.1 nmol/l (2.5 ng/ml) fluorescently labelled (NT-647) PDGF-BB. **A to C:** Various concentrations of PDGFRα-Fc wildtype and I139E (**A**), Y206S (**B**) or I139A + Y206A (**C**) were mixed with PDGF-BB. Dose dependent fluorescence quenching rates from 3 measurements were used to generate binding curves. The respective curves were fitted to a one site binding model to determine K_d_ values, graphed in **D**. Error bars indicate K_d_ confidence. **E**: No changes in the initial fluorescence were observed when PDGF-BB was mixed with the PDGFRα-Fc I139E + Y206S mutant. Therefore, the binding affinity for PDGF-BB was determined by microscale thermophoresis analysis. Binding curves were generated by analysis of the ratio (Δ F_norm_) of fluorescence at MST*-on* time (4–5) seconds over the steady-state fluorescence (F0) for each PDGFRα-Fc wild type or I139E + Y206S concentration. Error bars indicate standard deviation from 3 replicate measurements. **F** depicts the corresponding K_d_ values with confidence intervals. For comparison with other graphs in this manuscript please note that 0.14 nmol/l of PDGFRα-Fc correspond to about 24 ng/ml, while 200 nmol/l equal 33,000 ng/ml.

In order to determine binding affinities of PDGFs to PDGFRα-Fc wild type and the most promising mutants we initiated direct ligand binding experiments by microscale thermophoresis (MST). For this purpose, fluorescently labeled PDGF-BB (0.1 nmol/l = 2.5 ng/ml) was mixed with various concentrations of PDGFRα-Fc covering 5 log steps. Besides well-defined MST-traces, dose-dependent photobleaching effects were detected prior to the thermophoresis segment of the experiments particularly for PDGFRα-Fc wild type (Figs 7A–7C and S4A–S4D), indicative of a very strong interaction. Using this dose-dependent quenching signal, a K_d_ of 0.14 nmol/l was obtained for the wild type receptor molecule. The same K_d_ of 0.14 nmol/l was determined when the MST traces were chosen for analysis (compare Fig 7D and 7F) which demonstrates that both readouts are equally valid. When the single amino acid exchange mutants I139E and Y206S as well as the double alanine exchange mutant were tested by MST experiments, again a dose-dependent photobleaching effect was observed, although less pronounced compared to wild type PDGFRα-Fc. Corresponding analysis revealed 30- to 50fold decrease of the affinities to PDGF-BB with K_d_ values of 4.0, 7.3 and 4.6 nmol/l, respectively (Fig 7A–7D). However, the combination of I139E and Y206S mutations in PDGFRα-Fc did no longer cause any measurable change in PDGF-BB fluorescence, indicating a further, more drastic loss of affinity. Evaluation of this low affinity binding was accomplished by using the recorded MST-traces (Figs 7E and 7F, S2E and S2F). The resulting dose-response curve demonstrated that this PDGFRα-Fc double mutant interfered with the thermophoretic mobility of PDGF-BB only at high concentrations of more than 10 nmol/l (1,600 ng/ml). The corresponding K_d_ estimated from the dose response curve was 200 nmol/l, about 1,500fold higher than that of wild type PDGFRα-Fc.

Taken together, these experiments provide a proof of principle for increasing the selectivity of PDGFRα-Fc by combination of selected mutations in its ligand binding domain.

**Table 2:** Comparison of PDGFRα-Fc wild type and mutants regarding HCMV inhibition, PDGF affinity and the resulting selectivity.

	HCMV inhibition EC50 [ng/ml]^a^ (SEM)	PDGF binding K_d_ [nmol/l] (K_d_ confidence)^b^	Selectivity^c^ as compared to wild type
PDGFRα-Fc wild type	4.6 (± 0.7)	0.14 (± 0.03)	1
PDGFRα-Fc I139E	32.8 (± 8.3)	4.02 (± 1.51)	4
PDGFRα-Fc Y206S	31.1 (± 1.7)	7.32 (± 3.13)	8
PDGFRα-Fc I139E + Y206S	25.2 (± 1.3)	200 (± 73.7)	260

^a^100 ng/ml of PDGFRα-Fc correspond to 0.604 nmol/l.

^b^ K_d_ confidence describes the error of the fit. With a confidence of 68%, the Kd of the measured interaction is within the given range.

^C^ (K_d_ mutant/K_d_ wild type) / (EC50 mutant/EC50 wild type)

### Serum-induced cellular signaling is unaffected by treatment with the modified PDGFRα-Fc

**Fig 8:**
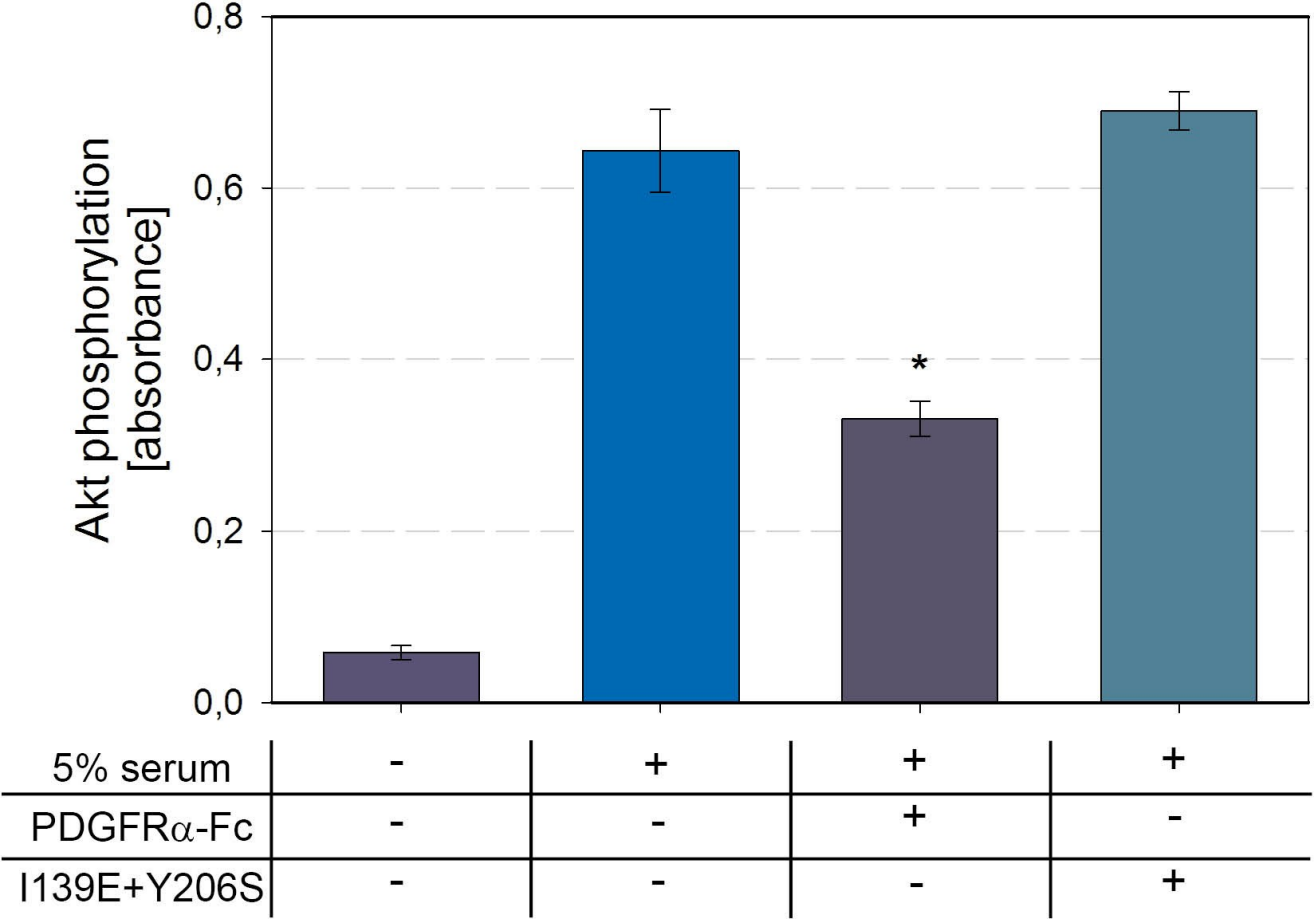
Serum induced cellular signaling is unaffected by treatment with the PDGFRα-Fc I139E + Y206S mutant. To test for cellular signaling under more physiological conditions, the effect of PDGFRα-Fc wild type and I139E + Y206S double mutant on serum-induced phosphorylation of Akt was measured. A pool of sera from 3 donors was preincubated with 562 ng/ml of soluble receptors before stimulation of fibroblasts. The absolute amount of Akt phosphorylation was measured using a commercial ELISA. Shown are the mean values for 3 experiments testing 2 independent protein preparations. The error bars indicate standard error of the mean. The asterisk indicates that only PDGFRα-Fc wild type but not the I139E + Y206S mutant significantly interfered with serum induced signaling as determined by unpaired t-tests comparing the response of untreated serum to the treated sera.

To get a picture of the effect of PDGFRα-Fc variants on the naturally induced cellular signaling, we tested the effect of PDGFRα-Fc wild type and I139E +Y206S on cellular signaling induced by human serum. For this, a pool of human sera from 3 different donors was diluted in serum-free cell culture medium to a final concentration of 5%. To test the effect of PDGFRα-Fc wild type and I139E + Y206S mutant, the serum pool was pretreated with 562 ng/ml of either PDGFRα-Fc soluble receptor for 2 hours before fibroblasts were stimulated for 15 min. The effect of serum with or without pretreatment was assessed using a phospho-Akt ELISA (Fig 8). Stimulation with 5% serum alone did induce a strong cellular response which was 50% reduced if the serum was pretreated with PDGFRα-Fc wild type. In contrast, no inhibition of cellular signaling was observed when the serum was pretreated with PDGFRα-Fc I139E + Y206S. Apparently, the PDGFRα-Fc double mutant did not sequester any of the growth factors contained in the serum pool to a measurable extent. This set of experiments demonstrates that combination of the two most promising amino acid exchanges identified in this study is sufficient to abolish interference of PDGFRα-Fc with cellular signaling.

### Mutation of V242K in PDGFRα-Fc variants further increases the specificity of the soluble receptor

**Fig 9:**
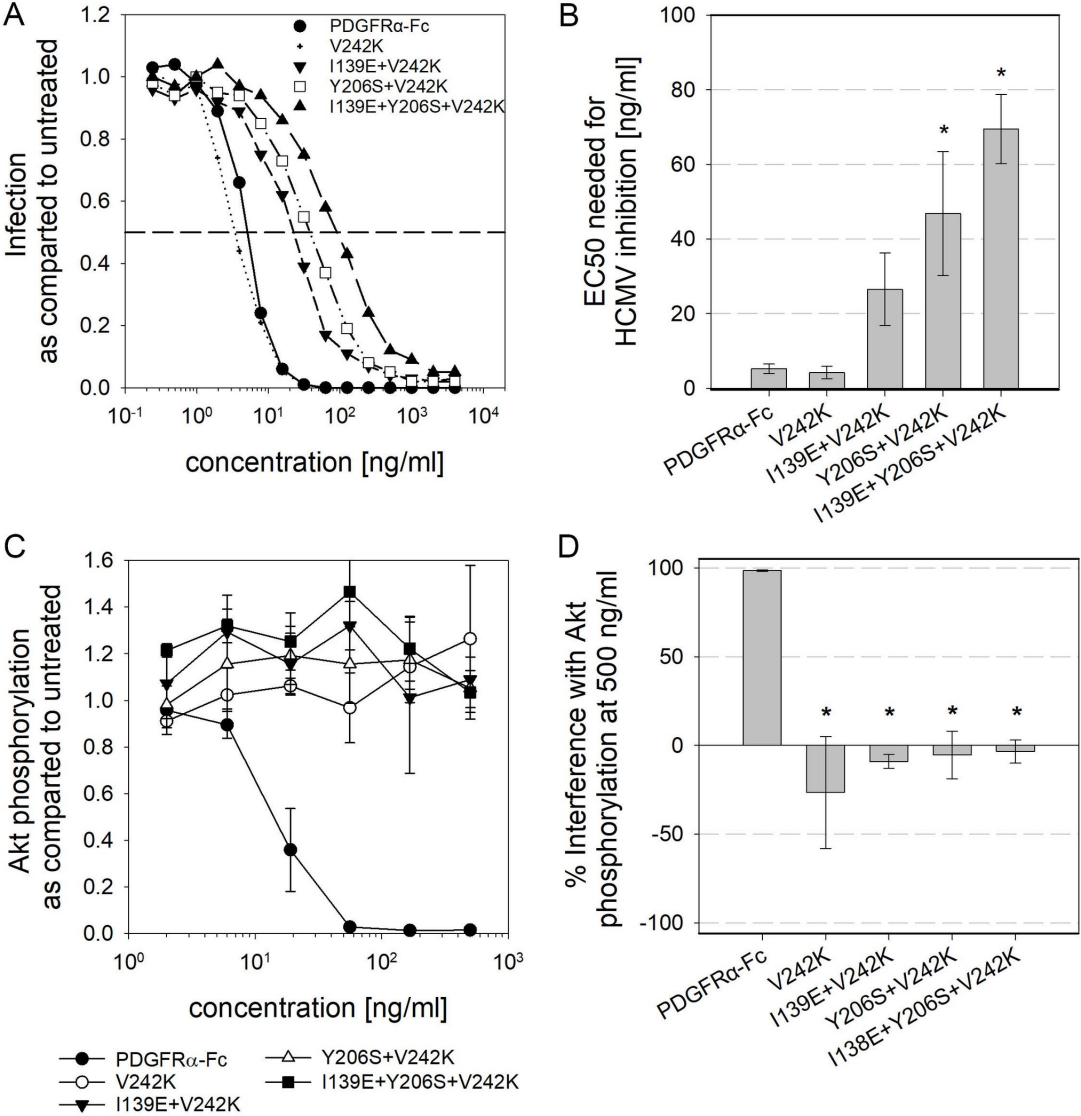
Mutation of V242K abolishes PDGF sequestration without impact on HCMV inhibition. **A + B:** Quantification of HCMV inhibition by PDGFRα-Fc V242K mutants. To measure the inhibition of infection, the rate of immediate early antigen-positive cells after infection with HCMV untreated or pretreated with PDGFRα-Fc was measured. **A** shows the average of 3 independent dose-response experiments and **B** shows the corresponding EC50 values. **C + D**: PDGF-dependent signaling after preincubation of 6 ng/ml PDGF-BB with various concentrations of PDGFRα-Fc mutants was quantified with a phospho-Akt ELISA. The dose-response curves shown in **C** depict the mean of 3 independent experiments and **D** compares the interference of 500 ng/ml of the different PDGFRα-Fcs with PDGF-dependent cellular signaling. 100% interference equals complete inhibition of Akt phosphorylation to the level of unstimulated cells whereas negative values indicate enhanced Akt phosphorylation as compared to cells that were stimulated with untreated PDGF-BB. All error bars indicate standard error of the mean.

**Fig 10:**
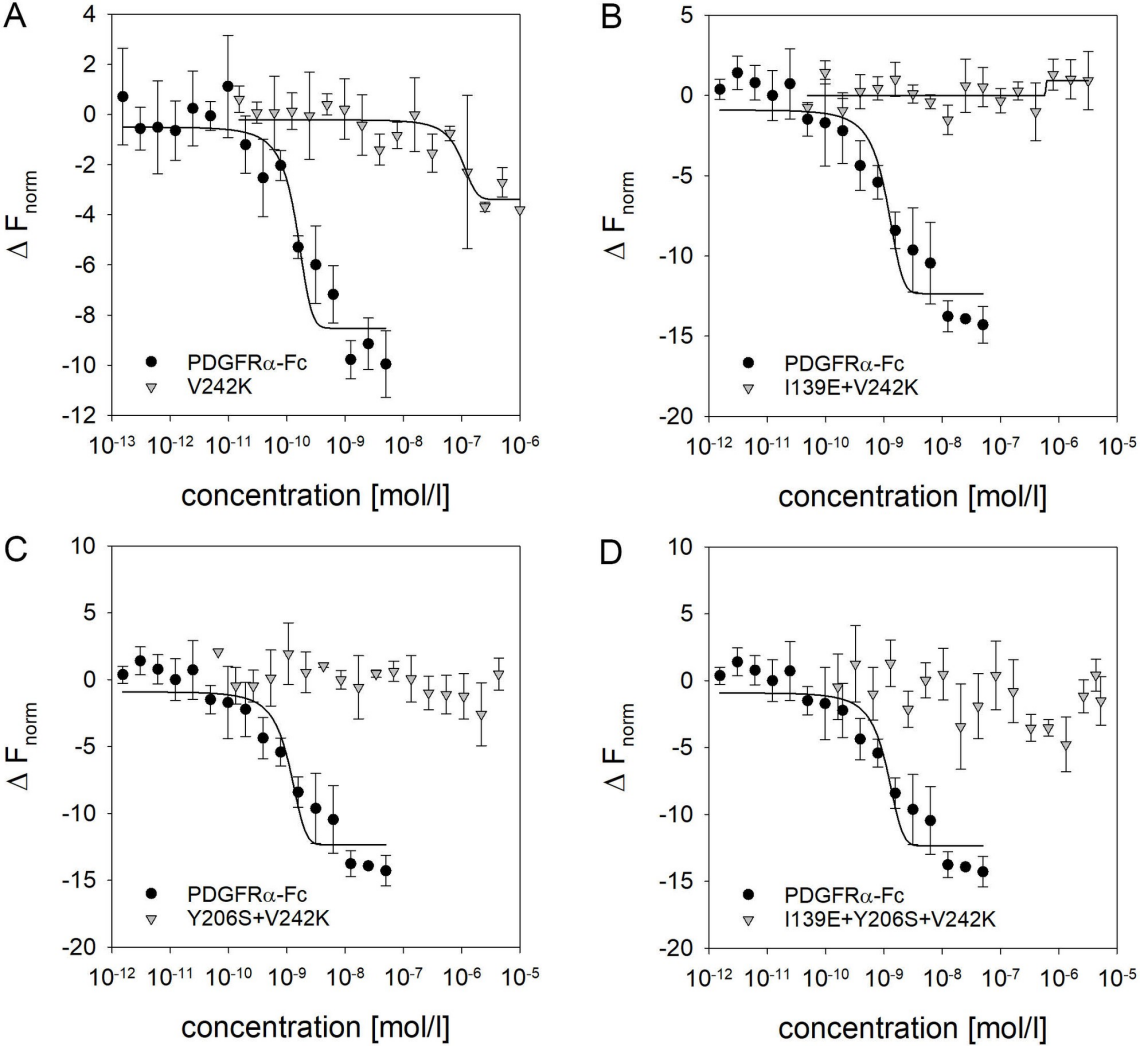
Combination of multiple mutations affecting PDGF binding reduces the affinity of PDGFRα-Fc for PDGF beyond detection. Quantification of biochemical binding affinity of PDGFRα-Fc variants for PDGF-BB was assessed by microscale thermophoresis. Various concentrations of PDGFRα-Fc wildtype, PDGFRα-Fc V242K (A), PDGFRα-Fc I139E + V242K (B), PDGFRα-Fc Y206S + V242K (C) or PDGFRα-Fc I139E + Y206S + V242K (D) were mixed with 0.1 nmol/l (**A**) or 1 nmol/l (**B to D**) fluorescently labeled PDGF-BB. Binding curves were generated by analysis of the ratio (Δ F_norm_) of fluorescence at MST*-on* time (1.5–2.5) seconds over the steady-state fluorescence (F0) for each concentration. Measurements were performed at 60% excitation power (**A**) or 20% excitation power (**B**). Error bars indicate standard deviation from 3 replicate measurements.

In a comprehensive screening approach recently described by Park et al. [50] the mutations Y206S and V242K were found most promising for abrogation of PDGF binding. As our previous experiments had demonstrated that combination of Y206S with I139E leads to strong reduction in PDGF binding affinity, the question was if combinations including V242K would further add to this effect. To this aim, a set of PDGFRα-Fc mutants was generated: a V242K single mutant, as well as combinations of V242K with I139E and Y206S. Like with the previous PDGFRα-Fc mutants, all of them were expressed as efficient as wild type and did not show any signs of degradation or aggregation (S1 Table and S5 Fig). After purification, these mutants were first tested regarding their ability to inhibit HCMV infection. In agreement with the data presented in Park et al. [50], the V242K mutation did not affect HCMV inhibition (Fig 9). Introduction of V242K into PDGFRα-Fc I139E or Y206S did also not significantly alter the efficiency of HCMV inhibition. The I139E + V242K mutant had an EC50 of 27 ng/ml as compared to 33 ng/ml for I139E, and Y206S + V242K caused half-maximal inhibition of HCMV at 47 ng/ml as compared to 31 ng/ml with Y206S. To test for sequestration of PDGF by the mutants, PDGF-BB was pretreated with different concentrations of the PDGFRα-Fc mutants (2–500 ng/ml) and the effect on PDGF-dependent signaling was assessed with a p-Akt ELISA. As expected, based on the results with the previous combination of mutations and the available information on V242K, none of the PDGFRα-Fc V242K mutations caused any reduction in Akt phosphorylation. To quantify the increase in specificity, the V242K mutations were included into the MST PDGF-BB binding experiments (Fig 10). Indicative of a strongly reduced affinity for PDGF-BB, no photobleaching was observed with the V242K mutant. Using 1000 nmol/l of PDGFRα-Fc V242K and 0.1 nmol/l of fluorescently labeled PDGF-BB, a K_d_ of 92 nmol/l was determined. Taken together with the unchanged efficiency for HCMV inhibition, this results in an 657fold increase in specificity as compared to wild type. Based on the pronounced reduction in binding affinity observed with the combination of I139E and Y206S, it was likely that the combinations of V242K with I139E and/or Y206S would decrease the affinity for PDGF-BB even further. Therefore, we adjusted the MST setup for higher MST sensitivity and used maximal available PDGFRα-Fc concentrations. Nonetheless, only for PDGFRα-Fc I139E + V242K a curve fit was possible, resulting in a roughly estimated K_d_ of 2,400 nmol/l. This would be close to a non-overlapping, additive effect of the two individual mutations. However, no distinct binding curves could be generated for any of the V242K combinations, indicating an extensive loss of binding affinity.

Taken together, these findings further emphasize the potential of combined mutations in PDGFRα-Fc, as a way to generate a soluble receptor which is inert to its natural ligand.

## Discussion

PDGFRα-Fc has been shown to be a promising inhibitor of HCMV infection [25,26,29,52]. However, using this decoy receptor against HCMV infection could also affect cellular signaling due to its ability to bind the natural ligands of PDGFRα. Our hypothesis-driven targeted approach demonstrates that (i) binding of PDGFs can be selectively abrogated by mutation of certain amino acids which reduce PDGF sequestration but are compatible with efficient neutralization of HCMV and ii) that combination of such mutations can additively reduce PDGF binding without reduction of HCMV inhibition, leading to a synergistic improvement of PDGFRα-Fc selectivity that prevents PDGF sequestration. These results can pave the way for further development of PDGFRα-Fc as an HCMV entry inhibitor.

Our data confirm the assumption of interference with PDGF signaling. As predicted, wild type PDGFRα-Fc reduced signaling in the phospho-Akt pathway both by human serum and PDGF-BB. PDGF-BB sequestration was tested at 6 ng/ml which is about the concentration of PDGF-BB in human blood [53–55]. At these sub-saturating concentrations of PDGF-BB, inhibition by PDGFRα-Fc was already pronounced at concentrations that were just sufficient for inhibition of HCMV. This highlights the necessity to identify mutations which increase the selectivity of the soluble receptor.

To this aim, our mutagenesis approach specifically targeted those sites that had been predicted to be involved in binding of PDGF-A and -B [47]. Thereby, crucial positions (I139 and Y206) were identified. Individual mutation of I139E or Y206S reduced the affinity for PDGF-BB by 30- and 50fold, respectively. In case of Y206S this reduction was already strong enough to prevent detectable sequestration in the phospho-Akt assay. HCMV inhibition was only 6fold reduced, demonstrating that these mutations are increasing the selectivity of the soluble receptor. Surprisingly, combination of the two mutations resulted in a significant reduction of PDGF binding, without a further decrease of HCMV inhibition, thereby increasing the selectivity of PDGFRα-Fc by 260fold. This strong increase of selectivity suggests that combined mutation of I139E + Y206S in PDGFRα-Fc renders this molecule inert with respect to human growth factors while maintaining its efficiency for HCMV neutralization.

A recently published study by Park et al. [50] identified Tyr 206 and additionally Val 242 as important sites for PDGF binding that can be mutated without loss of efficiency for HCMV inhibition. Park et al. [50] performed a comprehensive mutation of all positions in domains 2 and 3 of PDGFRα and screened in a high-throughput flow cytometry-based competition assay for binding of HCMV and PDGFs. In contrast, we tested deletion mutants for compatibility with HCMV inhibition, followed by a hypothesis-driven selection of site-specific mutations. This allowed us to generate a relatively small set of Fc-fusion constructs that we directly included into our quantifiable assays. However, our strategy failed to pick out V242K, possibly because the small deletion of N240-L243 caused a severe structural intolerance leading to a full loss of inhibition. Like the deep mutational approach, our strategy also identified Y206S for dissecting PDGF binding from HCMV inhibition. Our quantitative assessment of HCMV inhibition and affinity for PDGF-BB allowed to determine selectivity of PDGFRα-Fc for inhibition of the virus. Combining I139E and Y206S added clearly to a decreased ligand binding while surprisingly keeping its inhibitory potential. These disproportionate effects of combined mutations resulted in a 260fold increase of the selectivity index. Using these quantitative assays, we also investigated mutation of V242K. PDGFRα-Fc with this mutation was fully effective at blocking HCMV entry, but showed a strongly reduced affinity to PDGF, resulting in a 660fold increase of specificity. Combinations of I139E and/or V206S with V242K reduced the affinity to PDGF-BB even more, beyond our limit of detection, which is defined by the highest available PDGFRα concentrations (about 1 mg/ml). It can be assumed that interactions with affinities so low that they are not measurable at 1 mg/ml are highly unlikely to cause any biological interference. Even the least potent of the combination mutants, PDGFRα-Fc I139E + Y206S + V242K, fully blocked HCMV infection at 2000 ng/ml, while no binding to PDGF was observed even at the highest concentration tested (720,000 ng/ml = 4.35×10^−6^ nmol/l). This demonstrates that concentrations higher than those tested are neither necessary nor reasonable. Unexpectedly, PDGFRα-Fc Y206S alone and the combination with I139E both enhanced cellular signaling rather than reducing it when applied to cells at high concentrations, about 50fold above the EC50 for HCMV inhibition. This increase was not observed in combination with V242K. While the reasons remain unclear, enhanced signaling with Y206S and V242K has also been noted in Park et al., in this case it was most pronounced in combination with PDGF-AA [50]. Notably, PDGFRα-Fc I139E + Y206S did neither enhance nor reduce cellular signaling induced by human serum when used at lower concentrations that promote full neutralization of HCMV. Because all of the tested combinations of mutations in PDGFRα-Fc strongly increased the selectivity of the receptor, factors other than efficiency of HCMV inhibition, such as protein stability or frequency of resistance might help to decide which of them is the best basis for further development. For PDGFRα-Fc I139E + Y206S no changes in protein stability were detected. No differences in protein yield or other signs of aggregation were observed for any of the point mutants, which is a promising basis for further development of these soluble receptors.

The identification of mutations in PDGFRα-Fc that dissect binding to HCMV and PDGFs may also contribute to basic research regarding the exact role of cellular PDGFRα during virus entry. Results on whether HCMV binding induces PDGFRα phosphorylation have been conflicting. Soroceanu et al. have shown that HCMV binding induces PDGFRα phosphorylation and their experiments with chemical inhibitors indicated that it is important for infection [24]. In contrast, Wu et al. did not detect PDGFRα phosphorylation above background level and found that PDGFRα signaling is not necessary for infection [30,31]. It is now conceivable to knock out the wild type protein in the respective target cells of HCMV and replace it by a mutant that does no longer bind PDGFs. This will allow to address the role of PDGFRα-mediated signaling for various aspects of virus host cell interaction. The experiments performed in this study also open some interesting questions regarding HCMV biology. gO is highly variable, especially in the N-terminal part and we have demonstrated previously that conserved residues within the N-terminus of gO are important for PDGFRα engagement [42]. It is hence remarkable that PDGFRα-Fc I139E + Y206S was still capable of completely inhibiting various HCMV strains with distinct gO genotypes, albeit with different efficiencies. In contrast, certain gO isoforms were shown to be able to confer resistance to neutralization by anti-gH antibodies [56]. The differences that were observed for inhibition of diverse HCMV strains indicate that slight differences in the molecular interaction of the various gO-genotypes with PDGFRα exist. More detailed analyses of the individual mutations regarding their effect on the various HCMV strains may help to identify critical sites within both proteins that contribute to this interaction. It will be tempting to include also selected mutations from the recently published deep mutational approach [50].

For prevention of congenital CMV disease, several clinical studies have investigated anti-HCMV antibodies, either immunoglobulins derived from CMV seropositive individuals or recombinant monoclonal antibodies [35,37,57–60]. A recent study has found that frequent administration of HIG early during pregnancy significantly reduced transmission [57]. These findings confirmed previous studies that have shown lower disease severity or viral load by after HIG treatment [58,59]. These data strongly suggest that antibodies can be effective at reducing CMV disease. How much of these effects can be attributed directly to neutralization and which are the most important antigens is still a matter of debate [61,62]. Due to HCMVs ability to spread within tissues in a more protected, cell-associated way, antibody-dependent cellular cytotoxicity (ADCC) is assumed to play a role in restricting the virus. Interestingly, induction of ADCC has been shown for a peptide Fc fusion built with the same Fc fusion construct as PDGFRα-Fc [63]. This intriguing possibility should be investigated for PDGFRα-Fc and other soluble virus receptors in the future. The importance of cell-associated spread in the human host is still not understood due to the lack of a clear definition, aside of being antibody-resistant. While spread in fibroblasts cannot be blocked by antibodies [33,43,44], spread in epithelial and endothelial cells can [19,64,65], which seems to suggest that HCMV does not spread strictly cell-associated between these cells. Just like neutralizing antibodies, PDGFRα-Fc also does not block spread in fibroblast cultures. With regard to cell-free infection however, PDGFRα-Fc has several features that make it a promising alternative to monoclonal antibodies for prevention of HCMV transmission and disease. One of the most promising qualities of PDGFRα-Fc is its high potency for HCMV inhibition which is most likely due to the high affinity of the extracellular domain of PDGFRα for the viral gH/gL/gO trimer [25]. The efficiency of HCMV inhibition was slightly decreased with PDGFRα-Fc I139E + Y206S as compared to the wild type molecule, however with an EC50 of 25 ng/ml for fibroblasts its potency is still higher than what has been reported for antibodies that have been tested in clinical trials [32,36]. Importantly, PDGFRα-Fc inhibits infection of various cell types [25,26,29,52]. This is not the case for monoclonal antibodies against gB or gH, which are much less potent in neutralization of fibroblast infection than infection of epithelial cells [33,43,44,66–68]. A possible explanation for the difference between PDGFRα-Fc and anti-gB is that PDGFRα-Fc interferes with attachment of the virus as well as with penetration, whereas the anti-gB antibody clone C23 has been shown to only inhibit penetration [69]. Whether this difference in the mode of action correlates with the ability to inhibit infection of various cell types could be investigated in future studies. Because all HCMV strains rely on PDGFRα as their receptor for entry into fibroblasts it can be expected that PDGFRα-Fc is less prone to development of resistance by the virus. Future investigations should however address if development of resistance-conferring mutations is facilitated by mutations that affect PDGF binding. Also, the potential development of antibodies against PDGFRα-Fc needs to be considered, as this has been described for recombinant antibodies and other soluble receptors. Nonetheless, Fc fusion proteins are often discussed as a valuable alternative to antibodies. The first receptor-Fc fusion protein that has been licensed for therapy was Etanercept, a tumor necrosis factor (TNF) receptor Fc fusion that blocks TNFs to decrease inflammation in rheumatoid arthritis patients [70,71]. Recombinant ACE2 has already been shown to be safe in humans and has been discussed for treatment of SARS-CoV-2 patients with acute respiratory distress syndrome [72–74]). From the use of Etanercept and other Fc fusion proteins their generally long serum half-life (days to weeks) and good tolerability is well documented [75–77].

Taken together, the improved PDGFRα-Fc presented here is a promising molecule for neutralization of HCMV because it is can be expected to have desirable pharmacokinetics, it is most likely non-immunogenic, probably unlikely to induce resistance mutations, and it neutralizes infection of various cell types by various strains of HCMV. It is conceivable that PDGFRα-Fc alone or in combination with monoclonal antibodies may be favorable as compared to known antibody combinations. The identification of mutations which prevent interference with PDGFs pave the way for further development of this decoy receptor against HCMV infection.

## Materials and methods

### Human cells, sera and viruses

Primary human foreskin fibroblasts (HFFs) were obtained anonymized from residuals of routine procedures in agreement with the recommendations of the council of Europe (articles 21 and 23, year 2006). Propagation of the cells was performed in MEM supplemented with GlutaMAX (Life Technologies), 5% fetal bovine serum (FBS, PAN Biotech), 0.5 ng/ml basic fibroblast growth factor (bFGF) and 100 μg/ml gentamicin. For use in experiments the cells were kept in maintenance medium without bFGF. Serum starvation was performed in MEM with GlutaMAX but without serum or other supplements. Conditionally immortalized human endothelial cells [78], were cultured in endothelial cell growth medium (EGM bullet kit; Lonza) supplemented with 2 μg/ml doxycycline. All culture vessels were gelatine-coated for these cells. For experiments they were seeded in medium without doxycycline one day prior. Because of the inhibitory effect of heparin in the endothelial cell medium, the cells were preincubated with MEM for 30 min before infection. Human sera were obtained from healthy CMV-seronegative volunteer donors.

HEK 293 F suspension cells were propagated in 293 Freestyle medium (Thermo) supplemented with 100 μg/ml gentamicin under 8% CO2 and agitation at 130 rpm to a maximal density of 3×10^6^ cells/ml.

Most virological experiments were performed with HCMV TB40-BAC4-IE-GLuc, a BAC-cloned reporter virus based on strain TB40/E that expresses Gaussia luciferase under control of the major immediate early (IE) promotor/enhancer [48]. For comparison of PDGFRα-Fc with neutralizing antibodies regarding their dose response curves, HCMV strain TB40-BAC4 was used [79]. For analysis of the strain-dependency of inhibition by PDGFRα-Fc, HCMV strains AD169 [80], VHL/E [81], Towne [82] and Merlin [83] were used, which represent different genotypes of gO.

Virus stocks were harvested 5 to 7 days after infection of HFFs. The virus was cleared from cells and cell debris by centrifugation for 10 min at 2,700 × g. To additionally remove the luciferase released by the producer cells, the virus particles were ultra-centrifuged for 70 min at 100,000×g. The virus pellet was immediately resuspended in medium and stored at -80°C until used.

### Cloning of PDGFRα-Fc mutants

The extracellular domain (corresponding to aa 24 to 528) of PDGFRα (CD140a, NCBI reference number: XP_005265800) has been cloned into pFuse-hIgG1-Fc2 as described previously [42]. Mutagenesis primers were designed using the Agilent primer design tool, because the mutagenesis was performed using the QuikChange Lightning site-directed mutagenesis kit (Agilent). In short, the template plasmid was amplified with primers containing the desired mutation, then the template DNA was digested with Dpn I and transformed into XL10 chemically competent cells. Sanger sequencing of the PDGFR sequence was performed to exclude unwanted secondary changes.

### Expression and purification of soluble PDGFRα-Fc

Soluble PDGFRα-Fc fusion proteins were transiently expressed from HEK 293 cells. One day prior to transfection, 293 cells were diluted to 5×10^5^ cells per ml in gentamycin-free medium. For transfection, 1 μg of sterile DNA and 3 μg of polyethylenimine (PEI, 25K linear, Polysciences #23966) per 10^6^ cells were mixed in serum-free Opti-MEM (Thermo) and incubated for 15 min before the mixture was added to the cells. The transfection solution was incubated with the cells for 6 hours, before a medium exchange was performed. The PDGFRα-Fc containing medium was harvested at day 5 or 6 after transfection by pelleting the cells at 2,700×g for 10 min. To remove smaller cell debris, the supernatants were filtered through a 0.22 μm filter prior to concentrating the proteins with centrifugal filters with a pore size of 100kDa (Amicon). The PDGFRα-Fc fusion proteins were then purified using Protein A beads. The proteins were eluted from the beads with an elution buffer of pH 2.8 (Thermo #21004) and stored in the same buffer supplemented with 0.1 M Tris pH 8.0 at 4°C. The purified proteins were quantified photometrically measuring absorbance at 280 nm. For additional quality control, all protein preparations were checked by gel electrophoresis.

### HCMV inhibition assay

One day prior to infection, the cells were seeded on 96well plates at a density of 15,000 cells per well. The next day, HCMV TB40-BAC4-IE-GLuc virus stocks were diluted to obtain a final infection rate of about 50% in the untreated sample. These virus dilutions were then mixed with either only maintenance medium (untreated control) or with serial dilutions of PDGFRα-Fc proteins. The virus-inhibitor mixtures were incubated for 2 h at 37°C, before they were added to the cells for infection. One day later the percentage of inhibition was determined either by quantification of Gaussia-luciferase activity in the supernatants or by immunofluorescence staining of the cells. The use of Gaussia luciferase for quantification of infection has been described previously [48]. Briefly, the Gaussia luciferase-containing cell culture supernatants were either stored at -20°C or evaluated immediately in a plate reader (Hidex Chameleon) using coelenterazine (PjK GmbH) as a substrate for the luciferase. Coelenterazine was diluted to 0.2 μg/ml in phosphate buffered saline with 5 mM NaCl and added automatically to the cell culture supernatants in the plate reader to ensure timely measurement of the luminescence signal. The percentage of infection was calculated from the resulting luminescence signals by normalization to the untreated virus control. For comparison of the inhibitory effects of PDGFRα-Fc with human monoclonal neutralizing antibodies, virus was preincubated with a dilution series of the respective antibody and cell cultures were infected as described for PDGFRα-Fc. One antibody specific for glycoprotein B (clone C23) [84] and one antibody specific for the pentamer (clone 4I22) [33] were used. For quantification of infection by immunofluorescence, the cells were fixed with 80% acetone for 5 min at room temperature, before sequential incubation with an antibody against the viral immediate early antigens (E13, Argene) and secondary antibody goat anti-mouse IgG F(ab’)^2^-Cy3 (Jackson ImmunoResearch). Cell nuclei were stained with 4′,6-Diamidin-2-phenylindol (DAPI). Quantification was performed using Zen software (Zeiss).

### Quantification of Akt phosphorylation

#### Stimulation of cellular signaling

HFFs were seeded at a density of 100,000 cells per well in maintenance medium onto 24well plates. About 4 hours after seeding, when the cells had adhered to the plates, a medium exchange was performed and the FBS-containing medium was replaced by MEM without any additives. The cells were kept in serum- and growth factor-free medium for 1 day. Prior to stimulation of the cells, PDGF-BB (R&D # 220-BB) was diluted in MEM to 12 ng/ml and then mixed 1:1 with different concentrations of PDGFRα-Fc, resulting in a final PDGF concentration of 6 ng/ml. For stimulation with human sera, the sera were diluted to a final concentration of 5%. Ligand/Serum and receptor were preincubated at 37°C for 2 h. Then the mixture was added to the serum starved HFFs and incubated for 15 min at 37°C to allow signaling, cumulating in Akt phosphorylation. The stimulation was terminated by lysis of the cells for either immunoblot analysis or ELISA. Please note that the protocol described here was optimized for increased sensitivity. The first experiments using alanine exchange mutants (Fig 3) had been performed under slightly different conditions, using 50 ng/ml of PDGF-BB and stimulating the cells for 2 h.

#### Detection of Akt phosphorylation by immunoblot

The cells were lysed in Laemmli lysis buffer with β-mercaptoethanol on ice, then scraped off the plate and boiled for 10 min at 95°C. An equivalent of 28,500 cells was loaded onto 10% polyacrylamide gels and electrophoresis was performed in tris glycine SDS buffer. The proteins were transferred onto PVDF membranes in tris-glycine buffer with 15% methanol. Membranes were blocked with TBS plus 0.1% Tween and 5% milk powder before staining with rabbit anti-phospho-Akt (Cell Signaling #4060) and rabbit anti-actin (Sigma # A 5060). Horseradish peroxidase (HRP)-conjugated goat anti-rabbit (Santa Cruz #sc-2054) was used as secondary antibody. Visualization and quantification of the signals was performed with Super Signal West Dura Extended Duration substrate (Thermo) using FusionCapt Advance Solo (v.7 Vilber Lourmat). Phospho-Akt and actin signals were quantified from the same membranes to allow normalization of the Phospho-Akt signal to the corresponding actin signal in order to account for slight differences in loading.

#### Detection of Akt phosphorylation by ELISA

The PathScan Phospho-Akt1 (Ser473) Sandwich ELISA Kit (Cell Signaling #7160) was used for detection of Akt phosphorylation according to the manufacturer’s instructions. In short, the cells of each well were lysed with 150 μl of ice-cold cell lysis buffer containing 1 mM phenylmethylsulfonyl fluoride for 5 min on ice. Then the lysate was homogenized using a syringe before the insoluble parts were removed by centrifugation for 5 min at 18,800×g. The samples were stored at -80°C until the assay was performed. 100μl of undiluted sample per well was added to a microwell plate coated with phospho-Akt (Ser473) rabbit antibodies. After washing off unbound lysate, the samples were incubated subsequently with Akt1 mouse antibody and HRP conjugated anti-mouse Ig antibody. Detection was performed at 450 nm in a plate reader (Hidex Chamaeleon) after incubation of the sample with TMB substrate for 10 min.

### Quantification of binding affinities

Binding affinities were quantified using Monolith NT.115 Pico (NanoTemper Technologies) instrumentation and analysis was performed using MO.Affinity Analysis Software 2.1.3. To allow for fluorescence detection, PDGF-BB (R&D # 220-BB) was reconstituted in MST-labeling buffer NHS and labelled with NT-647 dye using the amine reactive labelling kit RED-NHS (NanoTemper Technologies). Labeled PDGF-BB was separated from excess dye by small scale SEC according to manufacturer’s instructions, using IgG-Elution buffer (Thermo #21004 neutralized with 0.1 M Tris pH 8.0). Addition of 0.025% Tween-20, 50 mM NaCl and the use of Premium Capillaries (NanoTemper Technologies) prevented aggregation and adhesion effects during MST experiments. High affinity measurements were carried out at constant PDGF-BB concentration of 0.1 nmol/l and 60% excitation power, while the PDGFRα-Fc variants were added in 16 different concentrations spanning 5log steps. For high affinity PDGFRα-Fc variants (WT, I139E, Y206S, I139A + Y206A) a dose-dependent photo-bleaching effect was observed. To increase the signal to noise ratio and signal amplitudes of the photo-bleaching readout, experiments were performed using a prolonged initial fluorescence detection segment (5 seconds) followed by a shortened MST measurement of 7 seconds. For low affinity experiments, MST setup was set to standard conditions (3 seconds initial fluorescence, 20 seconds MST ON-time) using 1 nmol/l PDGF-BB and 20% excitation power. Dissociation constants (K_d_) were calculated by curve fittings of either dose-dependent photo-bleaching rates (fluorescence change per second; WT, I139E, Y206S, I139A + Y206A) or dose-dependent changes of MST traces (difference between state F1 and F0; WT, I139E + Y206S and V242K variants). All experiments were performed three times, at 25°C.

### Statistics

Data sets were compared to test for significant differences using the t.test function of Excel. All tests were performed under two-sided hypotheses. Statistical significance was assumed for p ≤ 0.05.

## Supporting information

S1 Table Comparison of PDGFRα-Fc wild type and mutants regarding protein yield. Soluble PDGFRα-Fc fusion proteins were expressed from HEK 293 cells. The respective plasmid DNA was transfected into the suspension cells using polyethyleneimine. Five to six days post transfection PDGFRα-Fc was purified from the medium with Protein A. The purified proteins were quantified photometrically measuring absorbance at 280 nm. The table lists the protein yield of the latest expressions normalized to the number of transfected cells as μg of protein per million cells.
(DOCX)

S1 Fig Dose-response relationship between PDGF-BB and Akt phosphorylation. Serum-starved fibroblasts were incubated for 15 min with various concentrations of PDGF-BB before the cells were lysed. The PDGF-dependent signaling was assessed by immunoblot and staining for phospho-Akt. Actin was included as a loading control. **A** shows a representative example of such an immunostaining. **B** depicts the average dose-response in 4 independent experiments. Error bars indicate standard error of the mean.
(TIF)

S2 Fig Inhibition of different HCMV strains by PDGFRα-Fc wild type and I139E + Y206S mutant. Because the target of PDGFRα-Fc, the HCMV glycoprotein O, is a highly polymorphic protein, gO genotypes other than 1c of TB40 were tested regarding sensitivity to wild type PDGFRα-Fc and the I139E + Y206S mutant. For this, VHL/E BAC19 (gO 2b), AD169 (gO 1a), Towne (gO 4), and Merlin (gO 5) were diluted to an MOI of ≤ 1 and preincubated with different concentrations (0.07 to 4000 ng/ml) of the inhibitors for 2 h at 37°C before infection of fibroblasts. One day later the cells were fixed and stained for the viral immediate early antigens. Shown is the infection rate as compared to untreated virus. Dose response curves were generated as an average of 2 independent experiments.
(TIF)

S3 Fig Stability of PDGFRα-Fc. Using a NanoTemper Tycho T6 the protein folding and stability was tested. Tryptophane fluorescence was detected over a temperature gradient. All proteins were diluted to 50 μg/ml. **A:** Example of the fluorescence intensity ratio for PDGFRα-Fc wild type and I139S + Y206S. **B** shows the 330 nm detection of the same measurement. **C:** Comparison of two preparations of PDGFR α-Fc wild type, one after 1 month of storage at 4°C, the other after 1 year. **D:** Mean inflection temperatures of 3 measurements of PDGFRα-Fc and I139 + Y206S are shown. Error bars indicate standard deviation.
(TIF)

S4 Fig MST traces for PDGF-BB bound to PDGFRα-Fc variants. Microscale thermophoresis (MST) was performed with 0.1 nM (2.5 ng/ml) fluorescently labelled (NT-647) PDGF-BB which was mixed with different concentrations of PDGFRα-Fc wild type and mutants (2fold dilution series starting from **A + E**: 5nM, B: 500 nM, C: 650 nM, D: 550 nM or F:500 nM). In all graphs, the darkest line represents the highest concentration of soluble receptor and lightest grey depicts the lowest concentration. Each concentration was tested three times, shown is one example. All MST experiments were performed in the same way, with an initial fluorescence segment of 5 seconds, followed by a 7 second thermophoresis segment, at 25°C, with medium MST power and 60% excitation power. **A to D**: Direct fluorescence analysis measuring binding-induced fluorescence quenching for 5 seconds (white). MST traces are underlaid in green. The rates of fluorescence changes were used to generate dose-response curves shown in Fig 6A–6C. **E + F**: For thermophoresis analysis the ratio of fluorescence intensity after infrared laser activation (F1, red) over the initial fluorescence (F0, blue) was taken. Dose response curves shown in Fig 6E were calculated by MST traces shown here.
(TIF)

S5 Fig Electrophoretic mobility of reduced and non-reduced PDGFRα-Fc proteins. To control for dimer formation and degradation, 0.5 to 5 μg/ml of purified PDGFRα-Fc variants were either left untreated or treated with β-Mercaptoethanol. Proteins were loaded onto 4–20% Bis-Tris precast gels. For visualization of the proteins, a coomassie staining was performed.
(TIF)
