# Supplementary material for: Targeted mutagenesis on PDGFRα-Fc identifies amino acid modifications that allow efficient inhibition of HCMV infection while abolishing PDGF sequestration
Source: PLoS Pathog. 2021 Mar 29;17(3):e1009471. doi: 10.1371/journal.ppat.1009471 (PMC8031885; doi:10.1371/journal.ppat.1009471)
Supplement: S1 Table — Soluble PDGFRα-Fc fusion proteins were expressed from HEK 293 cells. The respective plasmid DNA was transfected into the suspension cells using polyethyleneimine. Five to six days post transfection PDGFRα-Fc was purified from the medium with Protein A. The purified proteins were quantified photometrically measuring absorbance at 280 nm. The table lists the protein yield of the latest expressions normalized to the number of transfected cells as μg of protein per million cells. (DOCX) [file ppat.1009471.s001.docx]

**Supplementary Table 1: Comparison of PDGFRα-Fc wild type and mutants regarding protein yield.**

|  | Protein yield [μg/10^6^ cells] | | | | |
| --- | --- | --- | --- | --- | --- |
|  | Expression 1 | Expression 2 | Expression 3 | Expression 4 | Expression 5 |
| PDGFRα-Fc wild type | 1.93 | 3.16 | 0.69 | 3.58 | 4.81 |
| PDGRα-Fc I139E | 1.65 | 3.44 | 1.51 |  |  |
| PDGRα-Fc Y206S | 2.34 | 1.93 | 1.1 | 1.93 |  |
| PDGFRα-Fc V242K |  |  |  |  | 3.44 |
| PDGFRα-Fc I139A+Y206A |  | 2.89 | 1.65 | 2.61 |  |
| PDGFRα-Fc I139E+Y206S |  |  | 0.96 | 2.89 | 6.60 |
| PDGFRα-Fc I139E+V242K |  |  |  |  | 3.99 |
| PDGFRα-Fc Y206S+V242K |  |  |  |  | 6.88 |
| PDGFRα-Fc I139E+Y206S+V242K |  |  |  |  | 4.54 |
