# Supplementary figures and images for: Targeted mutagenesis on PDGFRα-Fc identifies amino acid modifications that allow efficient inhibition of HCMV infection while abolishing PDGF sequestration
Source: PLoS Pathog. 2021 Mar 29;17(3):e1009471. doi: 10.1371/journal.ppat.1009471 (PMC8031885; doi:10.1371/journal.ppat.1009471)

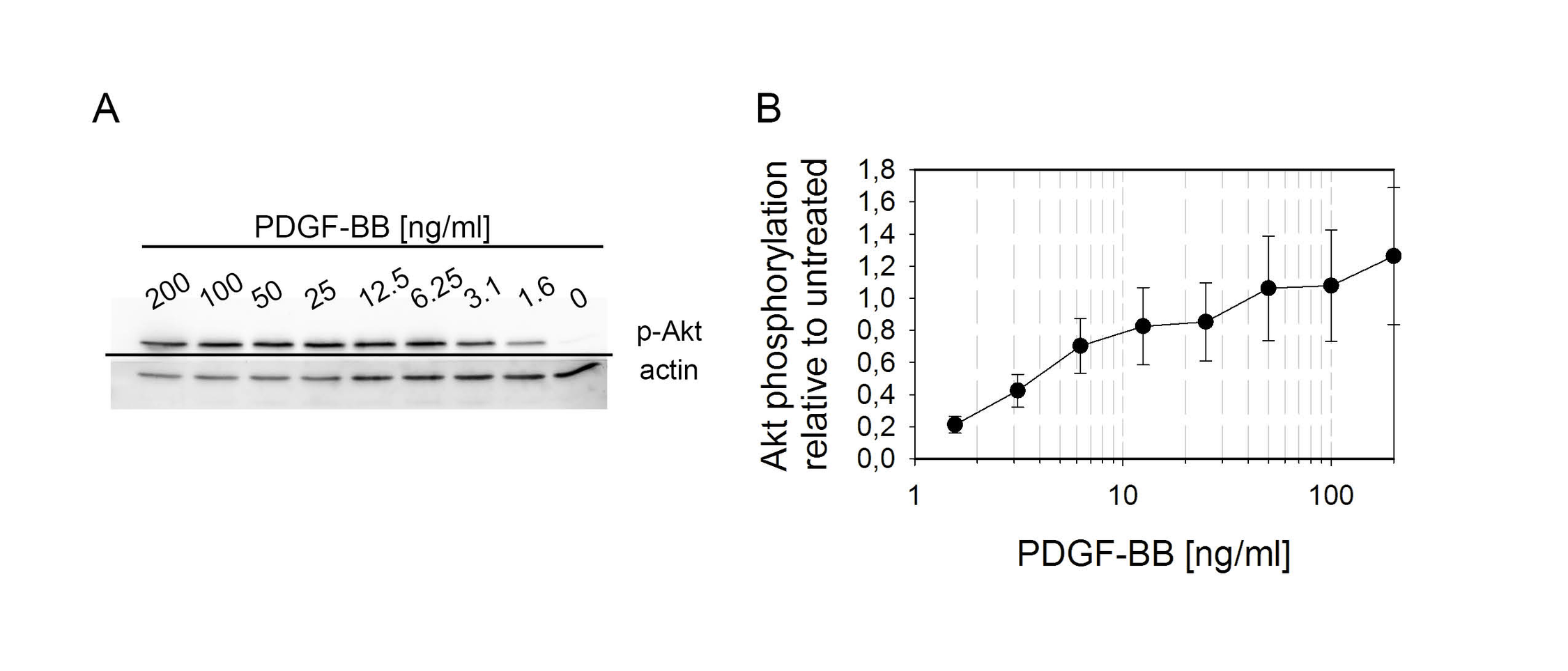

Supplement: S1 Fig — Serum-starved fibroblasts were incubated for 15 min with various concentrations of PDGF-BB before the cells were lysed. The PDGF-dependent signaling was assessed by immunoblot and staining for phospho-Akt. Actin was included as a loading control. A shows a representative example of such an immunostaining. B depicts the average dose-response in 4 independent experiments. Error bars indicate standard error of the mean. (TIF) [file ppat.1009471.s002.tif]

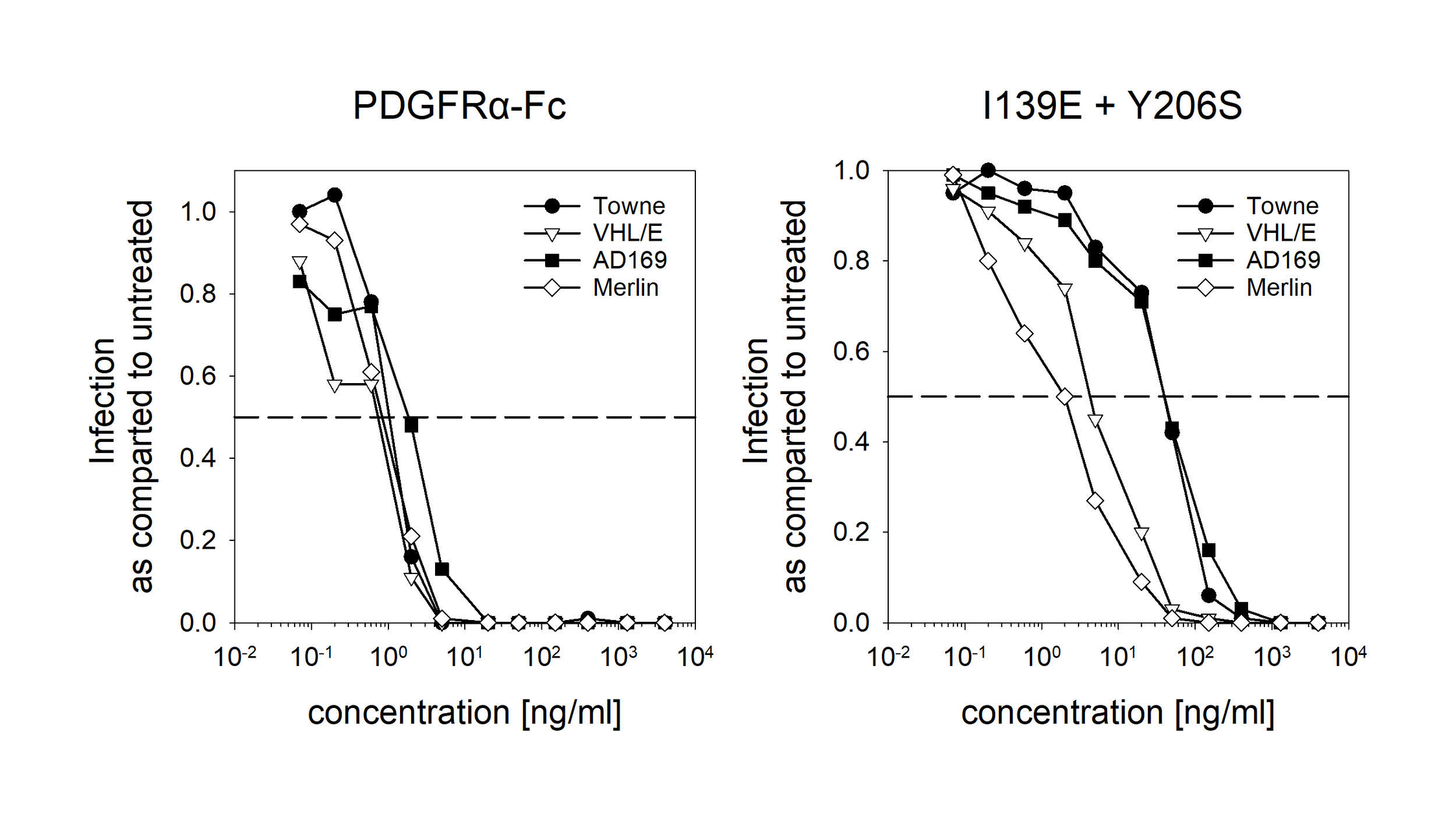

Supplement: S2 Fig — Because the target of PDGFRα-Fc, the HCMV glycoprotein O, is a highly polymorphic protein, gO genotypes other than 1c of TB40 were tested regarding sensitivity to wild type PDGFRα-Fc and the I139E + Y206S mutant. For this, VHL/E BAC19 (gO 2b), AD169 (gO 1a), Towne (gO 4), and Merlin (gO 5) were diluted to an MOI of ≤ 1 and preincubated with different concentrations (0.07 to 4000 ng/ml) of the inhibitors for 2 h at 37°C before infection of fibroblasts. One day later the cells were fixed and stained for the viral immediate early antigens. Shown is the infection rate as compared to untreated virus. Dose response curves were generated as an average of 2 independent experiments. (TIF) [file ppat.1009471.s003.tif]

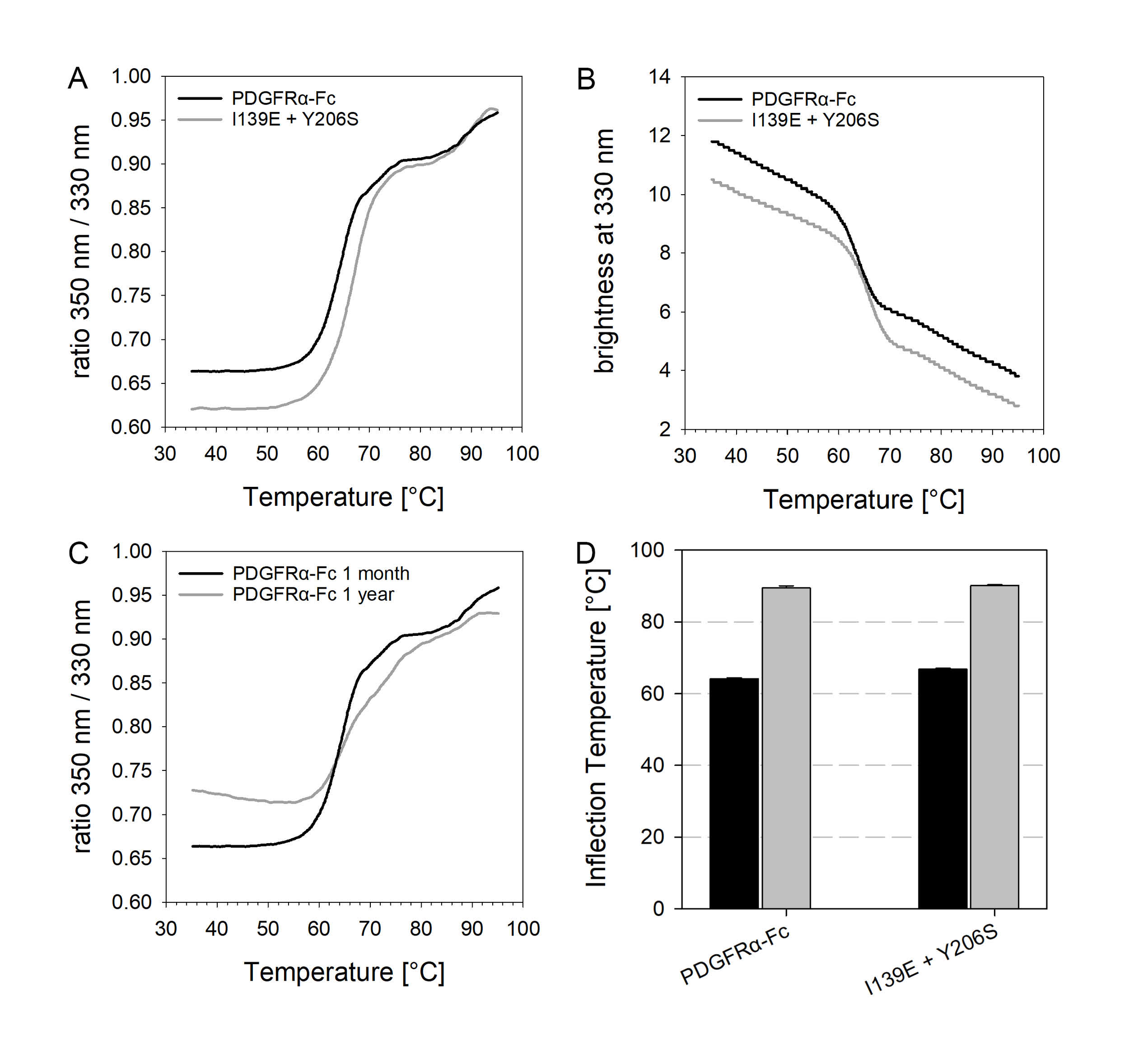

Supplement: S3 Fig — Using a NanoTemper Tycho T6 the protein folding and stability was tested. Tryptophane fluorescence was detected over a temperature gradient. All proteins were diluted to 50 μg/ml. A: Example of the fluorescence intensity ratio for PDGFRα-Fc wild type and I139S + Y206S. B shows the 330 nm detection of the same measurement. C: Comparison of two preparations of PDGFR α-Fc wild type, one after 1 month of storage at 4°C, the other after 1 year. D: Mean inflection temperatures of 3 measurements of PDGFRα-Fc and I139 + Y206S are shown. Error bars indicate standard deviation. (TIF) [file ppat.1009471.s004.tif]

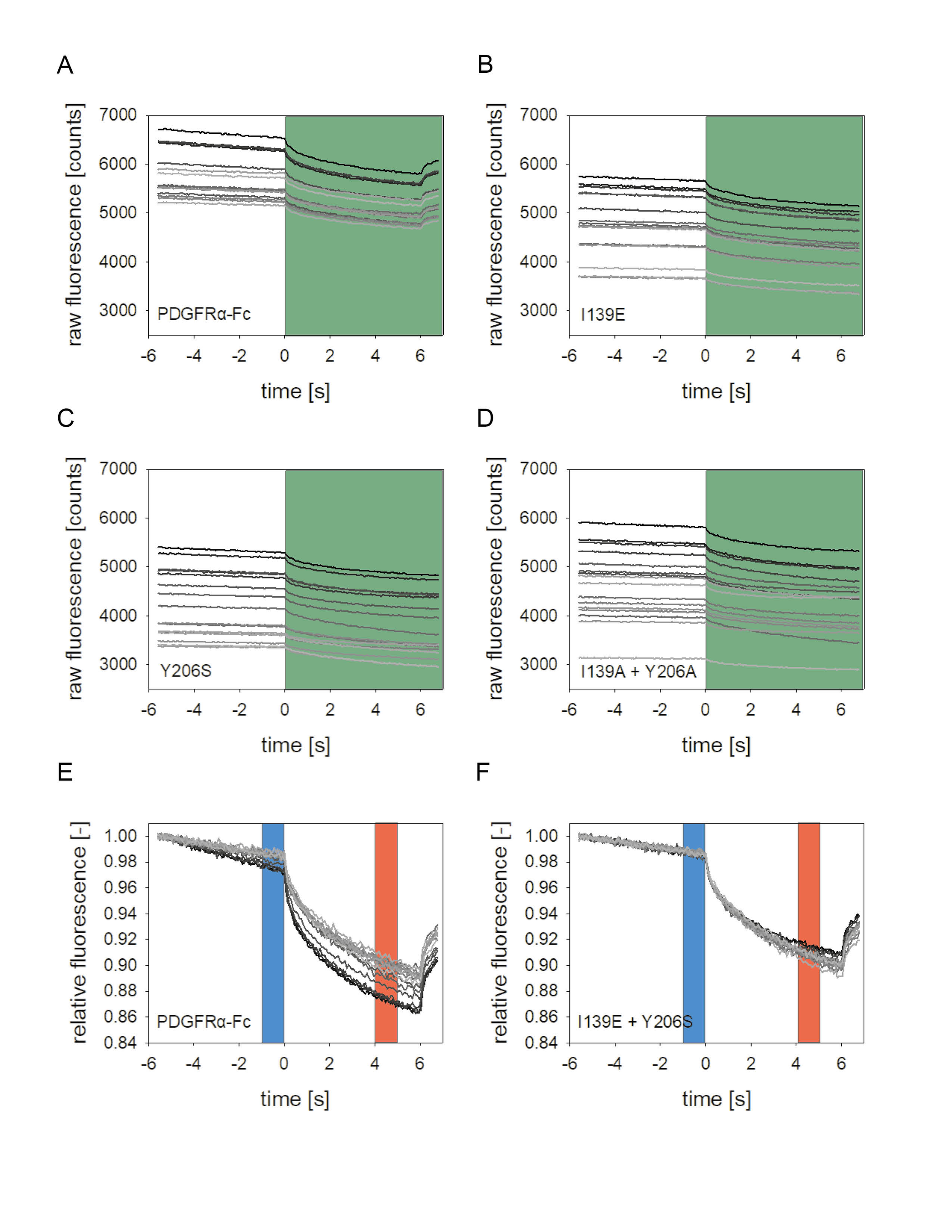

Supplement: S4 Fig — Microscale thermophoresis (MST) was performed with 0.1 nM (2.5 ng/ml) fluorescently labelled (NT-647) PDGF-BB which was mixed with different concentrations of PDGFRα-Fc wild type and mutants (2fold dilution series starting from A + E: 5nM, B: 500 nM, C: 650 nM, D: 550 nM or F:500 nM). In all graphs, the darkest line represents the highest concentration of soluble receptor and lightest grey depicts the lowest concentration. Each concentration was tested three times, shown is one example. All MST experiments were performed in the same way, with an initial fluorescence segment of 5 seconds, followed by a 7 second thermophoresis segment, at 25°C, with medium MST power and 60% excitation power. A to D: Direct fluorescence analysis measuring binding-induced fluorescence quenching for 5 seconds (white). MST traces are underlaid in green. The rates of fluorescence changes were used to generate dose-response curves shown in Fig 6A–6C. E + F: For thermophoresis analysis the ratio of fluorescence intensity after infrared laser activation (F1, red) over the initial fluorescence (F0, blue) was taken. Dose response curves shown in Fig 6E were calculated by MST traces shown here. (TIF) [file ppat.1009471.s005.tif]

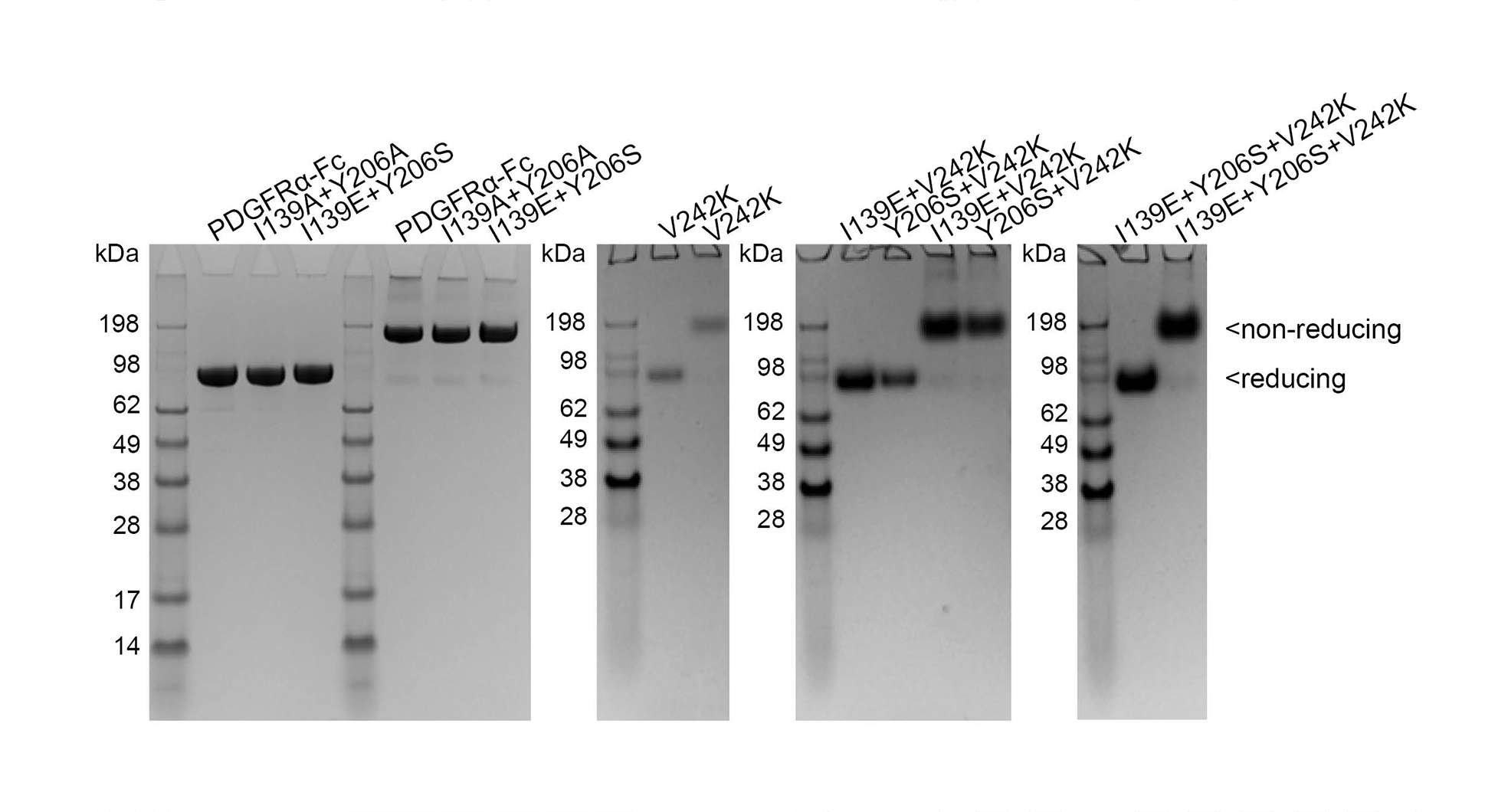

Supplement: S5 Fig — To control for dimer formation and degradation, 0.5 to 5 μg/ml of purified PDGFRα-Fc variants were either left untreated or treated with β-Mercaptoethanol. Proteins were loaded onto 4–20% Bis-Tris precast gels. For visualization of the proteins, a coomassie staining was performed. (TIF) [file ppat.1009471.s006.tif]
